# Exploring the potential of 5G uplink communication: Synergistic integration of joint power control, user grouping, and multi-learning Grey Wolf Optimizer

**DOI:** 10.1038/s41598-024-71751-2

**Published:** 2024-09-13

**Authors:** Sobana Sikkanan, Chandrasekaran Kumar, Premkumar Manoharan, Sowmya Ravichandran

**Affiliations:** 1 Department of Electronics and Communication Engineering, Karpagam College of Engineering, Coimbatore, Tamil Nadu 641032 India; 2 Department of Electrical and Electronics Engineering, Karpagam College of Engineering, Coimbatore, Tamil Nadu 641032 India; 3 Department of Electrical and Electronics Engineering, Dayananda Sagar College of Engineering, Bangalore, 560078 Karnataka India; 4 Department of Electrical and Electronics Engineering, Manipal Institute of Technology, Manipal Academy of Higher Education, Manipal, Karnataka India

**Keywords:** Competitive learning, Grey Wolf Optimizer, Non-orthogonal multiple access (NOMA), Q-learning, Spectral efficiency, User-grouping, Engineering, Mathematics and computing

## Abstract

Non-orthogonal Multiple Access (NOMA) techniques offer potential enhancements in spectral efficiency for 5G and 6G wireless networks, facilitating broader network access. Central to realizing optimal system performance are factors like joint power control, user grouping, and decoding order. This study investigates power control and user grouping to optimize spectral efficiency in NOMA uplink systems, aiming to reduce computational difficulty. While previous research on this integrated optimization has identified several near-optimal solutions, they often come with considerable system and computational overheads. To address this, this study employed an improved Grey Wolf Optimizer (GWO), a nature-inspired metaheuristic optimization method. Although GWO is effective, it can sometimes converge prematurely and might lack diversity. To enhance its performance, this study introduces a new version of GWO, integrating Competitive Learning, Q-learning, and Greedy Selection. Competitive learning adopts agent competition, balancing exploration and exploitation and preserving diversity. Q-learning guides the search based on past experiences, enhancing adaptability and preventing redundant exploration of sub-optimal regions. Greedy selection ensures the retention of the best solutions after each iteration. The synergistic integration of these three components substantially enhances the performance of the standard GWO. This algorithm was used to manage power and user-grouping in NOMA systems, aiming to strengthen system performance while restricting computational demands. The effectiveness of the proposed algorithm was validated through numerical evaluations. Simulated outcomes revealed that when applied to the joint challenge in NOMA uplink systems, it surpasses the spectral efficiency of conventional orthogonal multiple access. Moreover, the proposed approach demonstrated superior performance compared to the standard GWO and other state-of-the-art algorithms, achieving reduced system complexity under identical constraints.

## Introduction

The evolution of wireless communication technologies has been nothing short of transformative. From the early days of 1G to the current 5G era, each generation has brought forth significant advancements, shaping the way we communicate, work, and live. As we transition into the 5G era, the promise of ultra-reliable, low-latency communication, massive device connectivity, and enhanced mobile broadband beckons^1^. However, with these advancements come new challenges, especially in the realm of uplink communication. As we stand on the cusp of a fully interconnected world, the importance of optimizing uplink communication in 5G networks cannot be overstated^2^. Uplink communication refers to the transmission of data from a user device to a network and plays a pivotal role in ensuring a seamless user experience, especially in applications that require real-time data transmission. Uplink communication, the process through which data is sent from user devices to the network, is a critical component of any wireless system. In the dense, high-demand environment of 5G networks, ensuring efficient and reliable uplink communication becomes paramount. This necessitates innovative approaches to manage the myriad of devices and the vast amounts of data they transmit^3^.

Power regulation is one of the primary challenges in uplink communication. It is essential to make sure every device transmits at the best possible power level. While too little electricity can lead to lost or delayed data, too much power can interfere with other devices. Additionally, when the number of 5G devices increases as expected, individual management deteriorates. This introduces the idea of user grouping, a device clustering technique for more effective and economical resource distribution^4^. However, the integration of power control and user grouping, while essential, is not straightforward. It requires sophisticated optimization techniques to achieve the desired balance of efficiency and performance. The realm of 5G uplink communication presents a multifaceted environment with dynamic variables, making it a challenging domain to optimize^5^. Due to the following reasons, sophisticated optimization techniques are essential for this application. (i) Unlike static systems, 5G networks are characterized by their dynamic nature. Factors such as user mobility, fluctuating data traffic, and varying environmental conditions mean that the system's parameters are constantly changing. Simple optimization techniques that work for static or slowly changing systems may not be suitable for such a dynamic environment; (ii) The problem space in 5G uplink communication is massive. With numerous devices, each with its own set of parameters like power levels, transmission rates, and channel conditions, the optimization problem becomes high-dimensional. Sophisticated techniques are required to navigate this vast problem space efficiently; (iii) Many applications in 5G demand real-time or near-real-time responses. This necessitates optimization techniques that can provide solutions quickly, even in complex scenarios; and (iv) Achieving efficiency and performance often involves trade-offs^6,7^. For instance, maximizing data throughput might conflict with minimizing power consumption. Sophisticated optimization techniques can help in finding a balance between such conflicting objectives^8^.

Several optimization algorithms have been applied to the challenges of 5G uplink communication. For instance, a Genetic Algorithm (GA), inspired by the process of natural selection, is used for searching for optimal solutions by evolving a population of individual solutions^9^, Particle Swarm Optimization (PSO) is stimulated by the social conduct of birds flocking, PSO adjusts the trajectory of each individual solution based on its own experience and the experience of its neighbours^10^, Grey Wolf Optimizer (GWO) mimics the leadership hierarchy and hunting behaviour of grey wolves^11^. It has been applied to various optimization problems, including those in wireless communication and an area of machine learning where algorithms learn by interacting with an environment and receiving feedback in the form of rewards or penalties. Apart from the algorithms mentioned above, there are several other algorithms, namely artificial bee colony^12^, ant colony optimization^13^, differential evolution algorithm^14^, cuckoo search algorithm^15^, gravitational search algorithm^16^, firefly algorithm^17^, salp swarm optimizer^18^, whale optimizer^19^, marine predator algorithm^20^, equilibrium optimizer^21^, Slime Mould Algorithm (SMA)^22^, gradient-based optimizer^23^, Golden Jackal Algorithm (GJA)^24^, mountain gazelle optimizer^25^, thermal exchange optimizer^26^, red deer algorithm^27^, Aquila optimizer^28^, reptile search algorithm^29^, arithmetic optimization algorithm^30^, etc. are available in various literature. Many metaheuristics algorithms can sometimes converge to a solution too quickly before exploring the entire solution space, leading to sub-optimal solutions. Some algorithms tend to get stuck in local optima, meaning they find solutions that are optimal for a nearby region but not necessarily for the entire problem space^31,32^. Algorithms, especially deep reinforcement learning ones, can be computationally intensive, making them unsuitable for real-time applications. Many algorithms have parameters that need to be fine-tuned. The performance of the algorithm can be highly sensitive to these parameters, making it challenging to find the right set for a given problem. Some algorithms work well for small-scale problems but struggle to scale up to the size and complexity of real-world 5G networks.

Given these challenges and limitations, there is a pressing need for enhanced or new optimization techniques that can address the unique challenges posed by 5G uplink communication. In this context, drawing inspiration from the social hierarchy and hunting behaviour of grey wolves, GWO is a nature-inspired optimization method known for its efficacy in complex problem-solving^11^. The proposed Multi-Learning GWO (MLGWO) introduces several novel components specifically designed to address the limitations of the standard GWO, namely premature convergence and lack of diversity. These enhancements are achieved through the integration of Competitive Learning, Q-learning, and Greedy Selection mechanisms, each contributing uniquely to the overall performance of the optimizer. Competitive Learning is incorporated to balance exploration and exploitation during the optimization process. In this approach, multiple agents (grey wolves) compete to find the best solutions. This competition fosters a more diverse search process by preventing the optimizer from settling into local optima prematurely. The agents continuously adapt and improve their search strategies based on their performance relative to others, thereby maintaining a higher level of diversity in the population. Q-learning, a reinforcement learning technique, is utilized to guide the search process based on past experiences. By learning from previous iterations, the optimizer can better navigate the search space, avoiding redundant exploration of sub-optimal regions. This learning mechanism enhances the adaptability of the GWO, allowing it to adjust its search strategy dynamically and improve its chances of finding global optima. The Q-learning component helps the optimizer to remember and leverage beneficial moves while discouraging those that lead to premature convergence. The greedy selection mechanism ensures that the best solutions are retained after each iteration. This approach guarantees that the optimizer consistently builds upon the highest-quality solutions discovered, thus improving convergence speed without sacrificing solution quality. By focusing on retaining superior solutions, Greedy selection minimizes the risk of the optimizer getting stuck in sub-optimal regions, further mitigating premature convergence. This paper delves deep into the synergistic integration of power control, user grouping, and the enhanced GWO algorithm. Through rigorous research and testing, the study aims to present a comprehensive solution to the challenges of 5G uplink communication, setting the stage for a more connected and efficient future. The main contributions of this study are as follows.

- Introduction of MLGWO that integrates a multi-learning strategy which enhances the balance between exploration and exploitation, ensuring a more thorough and efficient search of the solution space.
- Model the objective function and constraints specifically tailored for 5G uplink communications. This involves accurately capturing the unique requirements and challenges of power allocation and user grouping in NOMA systems, enabling more effective optimization.
- Investigate user pairing and grouping in uplink NOMA transmission by examining specific predetermined power control approaches combined with near-perfect successive interference cancellation to provide insights into optimizing user allocation strategies to enhance spectral efficiency and overall system performance.
- Conduction of a detailed performance comparison of the proposed MLGWO with other state-of-the-art algorithms. Despite potentially higher complexity per iteration, the proposed approach demonstrates faster convergence and superior performance, as evidenced by the simulation results.

The paper is structured as follows. “Related works” section covers the related work and literature survey. “System modelling” section discusses in detail the system model and the objective function formulation. “Proposed algorithm” section discusses the formulation of the proposed mult-learning GWO algorithm along with the basic concepts of the GWO algorithm. “Results and discussions” section comprehensively discusses the obtained results, and “Conclusions” section concludes the paper.

## Related works

When examining spectral-power efficiency, NOMA methods have been confirmed to be more effective than Orthogonal Multiple Access (OMA) methods, particularly in fluctuating signal conditions. While OMA methods are simpler to implement, they are hindered by reduced spectral efficiency and scarce radio resources, making them less suitable for the extensive connectivity anticipated in upcoming network systems. The edge of NOMA over OMA arises from its ability to allow multiple users to share the same frequency channel within a single cell^33,34^. Among the various NOMA strategies, power-domain NOMA stands out, where users share the power domain. In such networks, Superpose Coding (SC) facilitates the blending of signals from different users on the same sub-carrier but with varied power intensities at the transmission end. Given that NOMA permits multiple users to utilize identical time, frequency, and code resources, it is inevitable that users encounter co-channel disruptions^35,36^. This necessitates intricate sub-band allocation methods and scheduling. Despite its advantages, NOMA systems demand significant computational resources, especially for Successive Interference Cancellation (SIC) and power distribution algorithms on the receiving end. The computational demands of SIC become even more pronounced when there is a need for high-speed data transfer and minimal delay^37,38^.

Recent research has explored the potential of integrating NOMA into standard protocols for IoT infrastructures and vehicle-to-everything communications. One of the hallmarks of NOMA systems is the use of the SIC method at the receiver side to enable the detection and decoding of multiple users. The application of the SIC method varies depending on whether it's for a downlink or an uplink, each having its unique operational sequence. In scenarios where multiple users are part of the NOMA cluster, there's a significant demand for computational resources^39^. As a result, SIC is typically applied in downlink NOMA scenarios. Conventionally, downlink NOMA configurations consist of pairs of users. Given the superior processing and computational abilities of the Base Station (BS), it is deemed feasible to employ SIC there for uplink NOMA systems. A notable benefit of uplink NOMA is its capacity to support concurrent transmissions from multiple users. However, to optimize the performance of NOMA systems while managing the computational demands of the SIC method, it is essential to develop innovative user pairing/grouping and power management tactics. The efficacy of both uplink and downlink NOMA configurations is closely tied to the chosen user pairing/grouping and power regulation methods^40,41^.

Cognitive radio technology enhances spectrum efficiency by allowing unlicensed users to access underutilized licensed spectrum bands opportunistically. Cognitive radios employ dynamic spectrum access techniques to identify and utilize spectrum holes, thereby improving overall spectrum utilization. However, NOMA offers several advantages over cognitive radio. NOMA can simultaneously serve multiple users in the same frequency band by exploiting power domain multiplexing, resulting in higher spectral efficiency without the need for extensive spectrum sensing and dynamic access protocols required in cognitive radio networks. Furthermore, NOMA's inherent capability to handle heterogeneous user demands and its compatibility with existing cellular infrastructure make it a more versatile and practical solution for enhancing spectral efficiency in modern wireless networks^42^. Integrated Sensing and Communication (ISAC) aim to integrate communication and sensing functionalities within the same wireless framework, thereby improving spectrum efficiency and enabling new applications such as vehicular networks and IoT. ISAC systems leverage joint resource allocation and cooperative strategies to optimize the dual functions of sensing and communication. While ISAC technologies present promising advancements, NOMA provides distinct advantages in terms of simultaneous user support and power domain multiplexing. NOMA's ability to dynamically allocate power levels to users based on their channel conditions and quality of service requirements allows for more flexible and efficient spectrum utilization. Additionally, NOMA's robustness in managing interference and its scalability to support massive connectivity make it a compelling choice for future wireless networks^43,44^. UAV-enabled integrated systems combine sensing, computing, and communication capabilities to enhance spectrum efficiency and enable advanced applications such as IoT and 6G communications. These systems involve joint resource allocation and trajectory design to optimize the overall performance. However, NOMA offers unique advantages by supporting multiple users simultaneously through power domain multiplexing, which can be further enhanced when integrated with UAV systems. NOMA's flexibility in power allocation and user grouping complements the dynamic nature of UAV deployments, leading to improved spectral efficiency and reduced latency^45,46^.

Numerous studies have delved into crafting effective user pairing/grouping strategies in NOMA systems with goals such as optimizing the overall rate, reducing transmission power, or ensuring equitable distribution. The researchers in one study proposed an innovative framework for devising suitable user pairing/grouping techniques to utilize the same resource blocks. Meanwhile, another study showcased a dual-user grouping approach rooted in distinct channel gains, with both types of research aiming to optimize the overall rate^47^. NOMA approaches surpass OMA systems in terms of spectral efficiency. Yet, to handle user signals as distinct units, a power control strategy at the transmission end is imperative. Additionally, various power distribution techniques are introduced for the standard single-cell uplink NOMA transmission framework. Power allocation for a single cell is noticeably simpler than that in a multi-cell scenario due to the interference between the channels. A viable solution to mitigate interference in multi-cell contexts is the adoption of a power-distributed downlink allocation strategy^38^. The authors of^48^ investigate the facilitation of Ultra-Reliable Low-Latency Communication (URLLC) systems through unmanned aerial vehicles (UAV)-enabled relaying for multi-access edge computing (MEC) systems in 6G networks. This study provides a forward-looking perspective on the integration of UAVs and MEC systems, emphasizing the role of URLLC in future networks. Their findings on resource allocation and relay positioning are particularly relevant to our research, as they address similar challenges in optimizing network performance and efficiency.

Research on resource allocation for NOMA is detailed in^49^, with a predominant emphasis on optimizing the combined rate while adhering to total power and proportional rate limitations. Additionally, multi-cell NOMA is explored in^50^. The authors of^51^ have suggested a solution nearing optimality for power distribution with the assumption of flawless Channel State Information (CSI) at the base station. Conversely, the authors of^50^ introduced a proficient power allocation method tailored for varying Quality-of-Service (QoS) needs, especially when CSI is not perfect. The primary objective of these studies was to curtail the overall transmission power. Moreover, the combined efforts of power distribution and subcarrier assignment for NOMA are discussed in^52–54^. The authors of^53^ focused on investigating the best power allocation to boost the weighted combined rate, given QoS restrictions. Specifically, the authors of^52^ showcased a near-best combined power and subcarrier distribution aimed at enhancing the weighted system output. Meanwhile, the authors of^54^ provided theoretical perspectives and a strategy for maximizing the combined rate. Yet, these methodologies primarily target either system output enhancement or total rate optimization, often side-lining user fairness, which is a pivotal aspect in shaping NOMA networks. In another relevant study, the authors of^55^ address the quasi-optimization of resource allocation and positioning for solar-powered UAVs. This research highlights the importance of resource allocation and efficient positioning strategies for enhancing the performance of UAV networks. The methodologies and optimization techniques discussed in this paper complement our approach to power control and user grouping, offering additional insights into resource management in next-generation networks.

In a study referenced in^56^, the authors explored the best power allocation for three unique uplink NOMA setups using the Hungarian method, adjusting the cost function related to user pairing techniques. They also developed various user pairing/grouping strategies based on matching games. Recent research delved into user-pairing and grouping methods for different NOMA systems. The authors of^33^ highlighted the significance of user grouping and power control for attaining high sum-rate capability, providing a lookup table derived from comprehensive calculations. The authors of^57^ introduced a resource allocation method for uplink scenarios based on the Cumulative Distributive Function (CDF), selecting the top two users based on the CDF's highest value. Meanwhile, research in^58^ presented a broad strategy for user categorization in overlapping NOMA networks, emphasizing machine learning to determine optimal user grouping. This research also tackled user grouping, affiliation, and power allocation, aiming to enhance the uplink network's capacity and overall system efficiency. The authors of^59^ explore power-efficient URLLC in UAV networks under jittering conditions. This study highlights the significance of power efficiency and reliable communication, which are critical factors for the effective deployment of UAV networks. Their work aligns with the focus on optimizing spectral efficiency and resource allocation in NOMA systems, providing a valuable perspective on power management in wireless communication networks.

In an early exploration of NOMA, uplink transmissions were examined in^60^, utilizing a power-control technique at the transmission end and employing a least mean squared error-based SIC process at the receiving end. The study in^34^ delved into the combined challenges of sub-carrier allocation and power distribution, ultimately deriving a near-optimal method to boost the spectral efficiency of NOMA participants. Diverging from prior research, the authors of^61^ introduced a resilient user-scheduling technique for NOMA uplink transmissions, capitalizing on the unique channel gains of network users and employing single-carrier frequency division multiple access to facilitate effective user grouping. For a two-user NOMA uplink transmission, the authors of^62^ formulated closed-loop solutions for sum outage and throughput probability under fixed power conditions. Additionally, if target sum rates are not properly chosen for NOMA downlink broadcasts, a comparison with time division multiple access-based OMA showed that a NOMA user could regularly experience an outage.

Metaheuristic algorithms are versatile optimization tools rooted in the principle of natural selection, making them adept at addressing diverse engineering challenges. They utilize a specific parameter to denote the intricacy of power control problems, formulating the function as a network's sum rate utility function^57,63,64^. As a result, all systems uniformly apply these power control parameters. Research has delved into NOMA-based mobile edge computing systems to enhance energy efficiency through task offloading. A coalition-based strategy has been employed to tackle power equilibrium and resource distribution^33^. Various meta-heuristic techniques were introduced to handle the localization challenges in wireless sensor networks, including the bat^65^, whale optimization^66^, firework^67^, and cuckoo search algorithms^68^. Additionally, a routing method leveraging a hybrid optimization algorithm has been developed for wireless sensor networks. Recently, swarm intelligence algorithms have gained traction as effective optimization tools in wireless communication^69,70^, standing alongside game theory^71^ and convex optimization^72^. Utilizing swarm intelligence algorithms can potentially resolve wireless network challenges, including power management, spectrum distribution, and security concerns. For optimal sum rate in NOMA downlink systems, GWO and PSO have been the focus. Furthermore, the research introduces a discrete version of the distributed GWO^73^ for the assignment of interdependent tasks to virtual machines. Within the framework, scheduling is perceived as a challenge with the primary objective of minimizing data movement and processing expenses. Recent advancements in global optimization have spotlighted the improved Grey Wolf Optimizer, addressing the limitations inherent in the original GWO algorithm^74^. The island-based cuckoo search represents a parallel adaptation of the traditional Cuckoo Search (CS) algorithm, incorporating the notably disruptive island CS polynomial mutation^75^. An algorithm with opposition-based learning was introduced for task scheduling in cloud computing environments, primarily focusing on data transfer costs and computational aspects^76^. Moreover, numerous iterative algorithms have been introduced to address energy efficiency optimization in NOMA networks, such as within NOMA HetNets^77^, a single-cell NOMA system^78^, and massive MIMO networks^79^. While the iterative method has been adapted to diverse contexts, the network configuration examined in this study is distinct, rendering the current solutions unsuitable. Specifically, if rigorous fairness guidelines are enforced to ensure equity among all users, the strategies presented in^77–79^ become inapplicable.

Th authors of^80^ propose a joint optimization framework for user grouping and power allocation in NOMA downlink systems to maximize spectral efficiency. By developing an efficient algorithm to solve this complex problem, the study demonstrates significant improvements in system performance through comprehensive simulations, addressing critical challenges in NOMA optimization. The authors of^81^ introduce a hybrid heuristic-based approach, combining genetic algorithm and particle swarm optimization, to optimize network parameters in NOMA systems, focusing on enhancing spectral and energy efficiency while ensuring QoS maximization. The integration of these heuristic algorithms effectively balances these objectives, with simulations showcasing superior performance compared to traditional methods. The study presented in^82^ proposed an improved Bat Algorithm to optimize throughput in NOMA cognitive relay networks incorporating RF energy harvesting. The novel system model and tailored optimization algorithm result in significant enhancements in throughput and energy efficiency, validated through detailed simulation results. The authors of^83^ propose the nonlinear marine predator algorithm for fair power allocation in NOMA Visible Light Communication (VLC) systems for B5G networks. By applying this bio-inspired algorithm, the study demonstrates improved performance and fairness in power allocation, offering a cost-effective solution for emerging VLC-based networks. The study presented in^84^ addresses resource allocation in downlink multi-channel NOMA systems, considering complex constraints such as interference and user mobility. The proposed efficient allocation scheme enhances system performance and robustness, as evidenced by comprehensive simulation results, making it relevant for practical deployment in NOMA networks. The study presented in^85^ explores the application of various meta-heuristic algorithms, including particle swarm optimization, differential evolution, satin bowerbird optimizer, symbiotic organism search, and artificial bee colony, for optimizing power allocation in indoor MIMO-NOMA VLC systems. By comparing the performance of these algorithms, the authors identify effective strategies for improving system capacity and energy efficiency, contributing to advancements in indoor communication technologies. The authors of^86^ develop a low-complexity heuristic algorithm to optimize power allocation and access mode selection in machine-to-machine networks. The algorithm balances performance with computational efficiency, showing enhanced network efficiency and reduced complexity through simulation results, which is crucial for IoT applications. The study presented in^87^ examines the optimization of resource allocation in NOMA-based mobile edge computing surveillance networks using genetic algorithm and particle swarm optimization. The dual optimization approach significantly enhances system performance, validated through simulations, and highlights the practical relevance for surveillance applications. The study presented in^88^ investigates power allocation in indoor NOMA VLC systems using meta-heuristic optimization algorithms, including genetic algorithm, particle swarm optimization, and differential evolution. By applying and comparing multiple algorithms, the research demonstrates substantial improvements in spectral efficiency and system performance, advancing the field of indoor VLC technologies. The authors of^88^ explore the use of various metaheuristic algorithms, such as genetic algorithm, particle swarm optimization, and ant colony optimization, for optimizing resource allocation in NOMA networks. Through a comparative analysis, the study identifies the most effective strategies, demonstrating significant performance enhancements in simulations, thus providing valuable insights for effective NOMA optimization.

## System modelling

This section details the basics of the uplink NOMA transmission system, the modelling of the NOMA uplink transmission system, and the objective function formulation. In addition, this section also discusses the solution to the selected problem.

### Uplink NOMA transmission

**Fig. 1:**
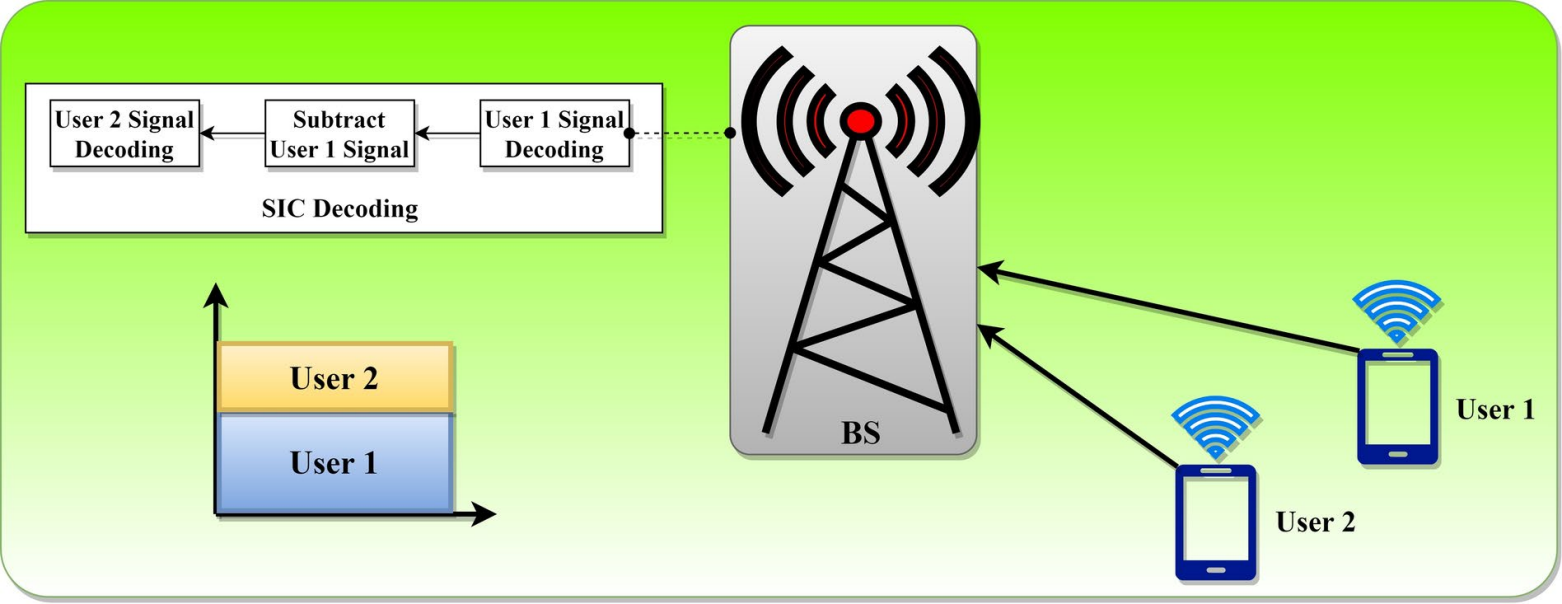
Illustration of uplink NOMA transmission for two users.

This segment delves into the idea of a two-user uplink NOMA transmission involving a singular BS. As depicted in Fig. 1, the uplink NOMA transmission's power-domain multiplexing is executed distinctively compared to the downlink NOMA transmission. This is because the BS utilizes superposition coding to achieve power province multiplexing in the downlink. In contrast, during the uplink, the broadcasting power of users is primarily constrained by their battery life. As a result, users can transmit at their maximum amount of power. The concurrent transmission of both users is represented by ${u}_{1}$ and ${u}_{2}$ to the appropriate base station^33,62^. The signals obtained from both users ${y}_{z}$ at the BS can be expressed as follows:

1 $${y}_{z}=\sum_{i=1}^{2}\sqrt{{P}_{i}}{g}_{i}{u}_{i}+{n}_{z}$$

where ${n}_{z}$ signifies the Gaussian noise, transmission power ${P}_{i}$ for ${u}_{i}$ with ${\mathbb{E}}|{({u}_{i})}^{2}|=1$, and the signal of the users $i(=\text{1,2})$. The signal ${u}_{1}$ is closer to the BS and is considered more robust, benefiting from a higher channel gain. In contrast, signal ${u}_{2}$ is weaker and situated further from the BS, resulting in a reduced channel gain. In uplink NOMA transmission, the SIC is utilized to decode a user's signal at the base station. As a result, the BS decodes signal ${u}_{1}$ first, treating signal ${u}_{2}$ as interference.

To decode signal ${u}_{2}$, the already decoded ${u}_{1}$ is subtracted, and then ${u}_{2}$ is decoded. In this uplink scenario, ${u}_{1}$ faces interference from ${u}_{2}$. However, transmission of ${u}_{2}$ is free from interference since ${u}_{1}$ is decoded before ${u}_{2}$. In the context of downlink NOMA transmission, the decoding process differs. The ${u}_{2}$ encounters interference from ${u}_{1}$, while transmission of ${u}_{1}$ is interference-free because ${u}_{2}$ is decoded before ${u}_{1}$. For the optimal functioning of SIC at the base station, the achievable data rates are denoted as ${R}_{i}$, with $i$ being either 1 or 2, for a specified bandwidth of 1 Hz^62^.

2 $${R}_{1}={\text{log}}_{2}\left(1+\frac{{P}_{1}|{g}_{1}{|}^{2}}{{P}_{2}|{g}_{2}{|}^{2}+w}\right)$$

3 $${R}_{2}={\text{log}}_{2}\left(1+\frac{{P}_{2}|{g}_{2}{|}^{2}}{w}\right)$$

For an efficient uplink NOMA system, users must have sufficiently distinct channel gains. If the channel gains of both users are too similar, the BS might struggle to distinguish their signals in the power domain, necessitating a robust power control strategy. This means users should transmit with varying power levels. Another crucial point from the earlier discussion is the reversed order of SIC in the uplink NOMA. In this setup, the signal of the user closer to the BS is decoded first, while in downlink NOMA, the past user's signal takes preference. Moreover, the BS typically has the capacity for enhanced optimization in terms of power and energy. As a result, uplink NOMA transmission can support more users than its downlink counterpart. The base station can also employ advanced decoding algorithms to reduce interference effects on users. Thus, for extensive machine-type communication, uplink NOMA is often more advantageous than downlink NOMA.

### Uplink NOMA transmission model

**Fig. 2:**
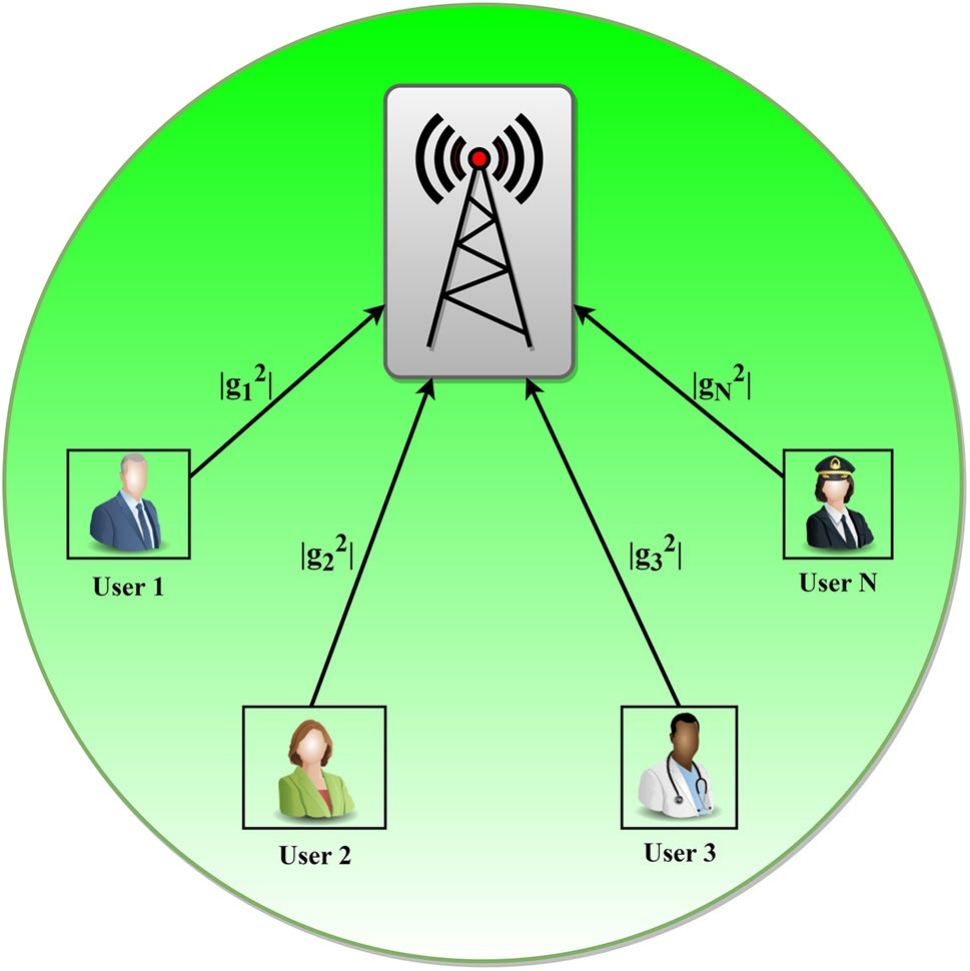
Illustration of the uplink NOMA transmission.

Consider an uplink transmission based on NOMA within a single cell, denoted as $C$, with $C$ being equal to 1. Within this cell, the BS is centrally located and facilitates communication with $N$ users, as illustrated in Fig. 2. It is assumed that both the base station and all $N$ users are equipped with one antenna. Additionally, the base station is fully informed of the CSI and caters to the users $N$ inside its service range. The total number of Physical Resource Blocks (PRBs), whether in terms of frequency, time, or code, is represented by $K$, with $K$ being less than $N$. Therefore, the distribution of $N$ is organized within $K$, ensuring each $N$ in the network meets the data rate condition. In the NOMA framework, users within the same PRB/group can send their PRB. In contrast, those in separate groups can transmit different PRBs in OMA^62^. Consequently, the BS can receive various $N$ signals, denoted as ${y}_{k}$, which can be mathematically expressed as follows:

4 $${y}_{k}=\sum_{n=1}^{N}{H}_{k,n}{u}_{n}+{n}_{k}$$

5 $${H}_{k,n}={U}_{k,n}{g}_{n}\stackrel{\sim }{{\alpha }_{n}}$$

6 $$\stackrel{\sim }{{\alpha }_{n}}=\sqrt{{\alpha }_{n}P},{\alpha }_{n}\in \left[\text{0,1}\right], \stackrel{\sim }{{\alpha }_{n}}\in \left[0,\sqrt{P}\right]$$

The overall transmission power and power control coefficient for all $N$ in the network are represented by $P$ and ${\alpha }_{n}$, respectively. The signal emanating from $N$ is denoted as ${u}_{n}(1 \le n \le N)$, with an expected value of ${\mathbb{E}}(|{s}_{n}{|}^{2})$ being 1. The channel response is Gaussian, characterizing the distribution between $N$ and the BS, and is symbolized by ${g}_{n}$. Meanwhile, the white Gaussian noise, having mean power, is represented by ${n}_{k}$ and ${\sigma }^{2}$ within the $k$th group^33,34,62^. The allocation of $N$ to the $k$ ^th^ group is symbolized by ${U}_{k,n}$ and it is represented as follows.

7 $${U}_{k,n}=\left\{\begin{array}{ll}1,& \quad \text{if the user }n\in groupk\\ 0,& \quad \text{otherwise}\end{array}\right.$$

8 $${H}_{k,n}=\left\{\begin{array}{ll}{g}_{n}\stackrel{\sim }{{\alpha }_{n}},& \quad \text{if the user }n\in groupk\\ 0,& \quad \text{otherwise}\end{array}\right.$$

For the decoding process, the BS utilizes SIC on every PRB to retrieve the intended information from users within a similar group. For user $n$, the sequence of decoding within the $k$th group or PRB is denoted by ${\Delta }_{k,n}$. Assuming ${\Delta }_{k,n}=a>0$ indicates that for every $n$, the decoding is the ath one in the $k$th group. Consequently, the spectral efficiency for each $n$ can be mathematically articulated as follows.

9 $${R}_{n}={log}_{2}\left(1+\frac{|{g}_{n}{|}^{2}{\alpha }_{n}\gamma }{\sum_{\genfrac{}{}{0pt}{}{j\ne n}{{\delta }_{k,j}>{\delta }_{k,n}>0}}^{N}|{g}_{j}{|}^{2}{\alpha }_{j}\gamma +1}\right)$$

In this context, the ratio of transmission power to noise is symbolized by $\gamma$, where $\gamma$ equals $P/{\sigma }^{2}$. Consider two users, $n$ and $j$, within a cell regarding their decoding sequence. The decoding of user $n$ precedes that of user $j$ in a group, depicted as ${\Delta }_{k,j}>{\Delta }_{k,n}$. It is presumed that the base station possesses superior processing capabilities and is completely informed, meaning it has complete CSI to decode signals or information efficiently. Furthermore, to handle user grouping and power control, every user $n$ in the network should transmit values ${\alpha }_{n}$ and ${U}_{k,n}$^62^. Thus, for each user $n$ that is part of the $k$th group, the spectral efficiency can be mathematically expressed as follows.

10 $${R}_{t}^{(k)}={\text{log}}_{2}\left(1+\sum_{{U}_{k,n}=1}|{g}_{n}{|}^{2}{\alpha }_{n}\gamma \right)$$

Equation (10) demonstrates that while each group is unaffected by the decoding order, individual user spectral performance inside a network is.

### Problem formulation

This study considers a comprehensive approach to address several interconnected challenges. Specifically, this study integrated the formulation of the power control issue with the task of grouping users and determining the sequence in which their signals are decoded. This integration was done while ensuring that all operations met the minimum requirements set for each component. To investigate deeper into the details: (i) Power control pertains to the management and allocation of power resources to ensure efficient communication. Proper power control is crucial to prevent interference, save energy, and maintain the quality of service; (ii) User grouping involves categorizing users into specific groups based on certain criteria. Effective user grouping can optimize resource utilization and enhance overall system performance; and (iii) In systems like NOMA, the sequence in which user signals are decoded can impact the overall efficiency and quality of the received signals. Therefore, determining the optimal order is crucial. All these components were addressed while ensuring that they adhered to their respective minimum requirements. For instance, every user, represented as $n$ in the network, has a baseline rate requirement for their data transmission. This rate is symbolized by ${r}_{n}$. Given these details, the objective is to enhance the spectral performance of the system. From the references^34,89^, this study formulated a problem statement that seeks to maximize this spectral performance.

11 $$\text{Maximize}{:} \; \{{U}_{k,n}\},\{{\Delta }_{k,n}\}\in \theta ,\{{\alpha }_{n}\}{R}_{t}=\sum_{k=1}^{K}{R}_{t}^{(k)}$$

Subjected to:

12 $$\left.\begin{array}{l}{C}_{1}:0 \le {\alpha }_{n} \le 1,\forall n\\ {C}_{2}:{R}_{n} \ge {r}_{n},\forall n\\ {C}_{3}:{U}_{k,n}\in \{\text{0,1}\},\forall k,\forall n\\ {C}_{4}:\sum_{k=1}^{K}{U}_{k,n}=1,\forall n\end{array}\right\}$$

The component ${\Delta }_{k,n}$ signifies the sequence in which the signals from different users are decoded at the base station. The order can influence the efficiency and clarity of the received signals. The variable $h$ encompasses all potential sequences or combinations in which users' signals might be decoded. This means that for a set of users, there are multiple ways their signals can be processed, and 'h' captures all these possibilities. The constraint ${C}_{1}$ pertains to the power with which signals are transmitted. It takes into account the maximum power limit or the upper bound for every user, denoted as $n$, within the network. Ensuring that transmissions stay within this power limit is crucial to prevent interference, conserve energy, and maintain signal quality. The constraint ${C}_{2}$ embodies the minimum data transmission rate that should be attainable for the system to function effectively. This rate is a benchmark to ensure that data is transmitted and received at a speed that meets the system's operational requirements. The constraints ${C}_{3}$ and ${C}_{4}$ play a role in user allocation. They act as markers or indicators to confirm that a user, represented as $n$, is correctly assigned to a specific group within the network. Proper user allocation is essential for efficient resource utilization and to ensure that each user's requirements are met within the system's framework. At the core, these components and constraints provide a structured framework to understand and optimize the system's operations, ensuring that signals are decoded efficiently, power is used carefully, data transmission rates are maintained, and users are appropriately grouped.

### Maximization of spectral efficiency for the uplink NOMA transmission systems

The problem dimensions, such as ${\alpha }_{n},{U}_{k,n}$, and ${\Delta }_{k,n}$ are intricately interconnected, which complicates the search for an optimal solution to the problem outlined in Eq. (11). Adding to the complexity, the variables associated with user grouping, ${\Delta }_{k,n}$ are of a combinatorial-integer nature. To address this, one should first tackle the combinatorial aspect of the power/control problem and subsequently determine the decoding sequence of the user. Once these are set, an effective strategy for user-grouping can be implemented^62^. When a fixed user-grouping scheme is in place, the value of ${U}_{k,n}$ remains independent across all unique groups, irrespective of the decoding order and power control considerations.

13 $$\{{\Delta }_{k,n}\}\in \theta ,\{{\alpha }_{n}\}{R}_{t}(k)$$

14 $${R}_{n}\ge {r}_{n},n\in {\mathcal{N}}_{k} \;\; \text{and} \;\ ;0\le {\alpha }_{n}\le 1,n\in {\mathcal{N}}_{k}$$

where ${\mathcal{N}}_{k}$ denotes the possible combinations of the $k$th PRB.

#### Method of decoding order

To execute the SIC process, the receiver decodes all signals of all users in a sequence determined by their channel conditions, starting from the strongest to the weakest. For instance, in the context of NOMA uplink, users with superior channel conditions are decoded at the base station earlier than others. Conversely, those with subpar channel conditions are decoded later. As a consequence, the durable user encounters intrusion from other network users, while the weak user experiences no interference. For the most efficient decoding sequence, as mentioned in^89^, the users, denoted as $N$, within a similar PRB or group are organized based on ${O}_{n}$. The sequence in which each user's signal is decoded is influenced by the power control strategy, leading to various potential feasible areas, which can be described as follows:

15 $${O}_{n}=|{g}_{n}{|}^{2}(1+\frac{1}{{\psi }_{n}})$$

16 $${\psi }_{n}={2}^{{r}_{n}}-1$$

From now on, Eqs. (15), (16) denotes that any user $n$ is decoded first as per the largest ${O}_{n}$.

#### Uplink power control

To handle the challenge of power control, this study turns to optimization techniques, specifically leveraging the principles of linear programming. Linear programming is a mathematical method used to find the best possible outcome in a given mathematical model for some list of requirements represented as linear relationships. In this study, the linear programming approach allows us to systematically adjust power levels to achieve optimal performance while adhering to certain constraints. The power control optimization is crucial to ensure efficient communication, minimize interference, and optimize energy consumption. By framing this as a linear programming problem, it is possible to determine the optimal power levels for each user or device in the network, ensuring that the overall system operates at its peak efficiency^62^. The mathematical representation of this power control optimization problem is detailed as follows:

17 $$P1: \{{\alpha }_{j}\}\sum_{j=1}^{M}|{g}_{j}{|}^{2}{\alpha }_{j}\gamma$$

Subjected to:

18 $$\left.\begin{array}{c}{C}_{1}:0\le {\alpha }_{j}\le 1,\forall j\\ {C}_{2}:|{g}_{j}{|}^{2}{\alpha }_{j}\gamma \ge {\psi }_{j}\left(\sum_{k=j+1}^{M}|{g}_{k}{|}^{2}{\alpha }_{k}\gamma +1\right),\forall j\end{array}\right\}$$

In this context, ${\alpha }_{j}$ represents the power control coefficient, with $j$ belonging to the set $\{1, 2, 3, ... c, M\}.$ Both Eqs. (16–18) exhibit linearity and are articulated using SNR (Signal-to-Noise Ratio) formulations. Consequently, the constraint 2 can be detailed as follows.

19 $$|{g}_{j}{|}^{2}{\alpha }_{j}\gamma -{\psi }_{j}\left(\sum_{k=j+1}^{M}|{g}_{k}{|}^{2}{\alpha }_{k}\gamma +1\right)\ge 0$$

Equation (19) is represented in matrix form as follows.

20 $$\gamma \left[\begin{array}{cccccc}|{g}_{1}{|}^{2}& -{\psi }_{1}|{g}_{2}{|}^{2}& -{\psi }_{1}|{g}_{3}{|}^{2}& \cdots & \cdots & -{\psi }_{1}|{g}_{m}{|}^{2}\\ 0& |{g}_{2}{|}^{2}& -{\psi }_{2}|{g}_{3}{|}^{2}& \cdots & \cdots & -{\psi }_{2}|{g}_{m}{|}^{2}\\ \vdots & 0& |{g}_{3}{|}^{2}& \cdots & \cdots & -{\psi }_{3}|{g}_{m}{|}^{2}\\ \vdots & \vdots & 0& \cdots & \cdots & \cdots \\ \vdots & \vdots & \vdots & \vdots & \vdots & \vdots \\ \vdots & \vdots & \vdots & \cdots & \cdots & |{g}_{m}{|}^{2}\end{array}\right]\left[\begin{array}{c}{\alpha }_{1}\\ {\alpha }_{2}\\ {\alpha }_{3}\\ \vdots \\ \vdots \\ {\alpha }_{m}\end{array}\right] \ge \left[\begin{array}{c}{\psi }_{1}\\ {\psi }_{2}\\ {\psi }_{3}\\ \vdots \\ \vdots \\ {\psi }_{m}\end{array}\right]$$

21 $$Set{a}_{i,j}=\left\{\begin{array}{cc}0& i>j\\ |{g}_{i}{|}^{2}& i=j\\ -{\psi }_{i}|{g}_{j}{|}^{2}& i<j\end{array}\right.$$

Then, Eq. (20) can be articulated as follows.

22 $$A\alpha \ge b \text{ with } \alpha =\left(\begin{array}{c}{\alpha }_{1}\\ \vdots \\ {\alpha }_{M}\end{array}\right) \text{and }b=\left(\begin{array}{c}{\psi }_{1}/\gamma \\ \vdots \\ {\psi }_{M}/\gamma \end{array}\right)$$

In conclusion, the problem can be reformulated as follows.

23 $$P1: {C}^{t}x$$

Subjected to:

24 $$\left.\begin{array}{c}{C}_{1}:A\alpha \ge b\\ {C}_{2}:0 \le \alpha \le 1\end{array}\right\}$$

where, $${C}_{i}=(|{h}_{i}{|}^{2}\gamma )$$.

The optimization challenge, labelled as $P1$, regarding power control, can be depicted in the conventional format used for linear programming. As a result, ${C}_{1}$ can be articulated subsequently:

25 $$\sum_{j=1}^{M}{a}_{i,j}{\alpha }_{j} \ge {b}_{i},\forall i\in \{\text{1,2},3,\dots c,M\}$$

Equation (26) is the standard format of Eq. (25).

26 $$\sum_{j=1}^{M}{a}_{i,j}{\alpha }_{j}-{S}_{i}={b}_{i},{S}_{i} \ge 0\forall i\in \{\text{1,2},3,\dots c,M\}$$

where ${\alpha }_{i} \le 1,\forall i\in \{\text{1,2},3,\dots c,M\}$ and ${\alpha }_{i} \ge 0$, ${t}_{i}$ denotes the slack parameters and ${S}_{i}$ denotes the excess parameter, therefore ${\alpha }_{i}+{t}_{i}=1,\forall i\in \{\text{1,2},3,\dots c,M\}$. Lastly, the usual form of Eq. (24) is as follows.

27 $$A\alpha -IS=b$$

28 $$\left[\begin{array}{cccc}{a}_{11}& {a}_{1M}& -1& 0\\ {a}_{M1}& {a}_{MM}& 0& -1\end{array}\right]\left[\begin{array}{c}{\alpha }_{1}\\ \vdots \\ {\alpha }_{M}\\ {s}_{1}\\ \vdots \\ {s}_{M}\end{array}\right]=\left[\begin{array}{c}{b}_{1}\\ \vdots \\ {b}_{M}\end{array}\right]$$

29 $$\left[\begin{array}{cccc}1& 0& 1& 0\\ 0& 1& 0& 1\end{array}\right]\left[\begin{array}{c}{\alpha }_{1}\\ \vdots \\ {\alpha }_{M}\\ {t}_{1}\\ \vdots \\ {t}_{M}\end{array}\right]=\left[\begin{array}{c}1\\ \vdots \\ 1\end{array}\right]$$

From now on, Eqs. (28) and (29) can be reformulated as follows.

30 $$\left[\begin{array}{ccc}A& -I& I\end{array}\right]\left[\begin{array}{c}x\end{array}\right]=\left[\begin{array}{c}{b}^{1}\end{array}\right]$$

31 $$Set{a}_{i,j}=\left\{\begin{array}{cc}0& i>j\\ |{g}_{i}{|}^{2}+1& i=j\\ -{\psi }_{i}|{g}_{j}{|}^{2}& i<j\end{array}\right. \;\; \text{and} \;\; I=\left[\begin{array}{cc}1& 0\\ 0& 1\end{array}\right],{b}_{i}^{1}={b}_{i}+1$$

Lastly, the optimization problem can be reformulated as follows.

32 $$P1: {C}^{t}x$$

Subjected to:

33 $$\left.\begin{array}{c}{C}_{1}:Bx=y\\ {C}_{2}:x \ge 0\end{array}\right\}$$

where $${C}^{t}=(-|{g}_{i}{|}^{2}\gamma ,for \;i=\text{1,2},\dots c,M)$$, $${C}_{i}=0,for\;i=M+1,\dots c,3M$$, and $$x=\{{\alpha }_{1},{\alpha }_{2},\dots c,{\alpha }_{m},{S}_{1},{S}_{2},\dots c,{S}_{m},{t}_{1},{t}_{2},\dots c,{t}_{m}\}$$.

#### User grouping/pairing

Developing an efficient algorithm that requires minimal computational time for user pairing or grouping is pivotal for a high-performing NOMA uplink system. Addressing this, a new variant of the GWO algorithm is introduced to tackle the inherent complexity. It has been explored to derive both optimal and near-optimal solutions for the user pairing/grouping challenge, with the ultimate goal of enhancing overall system performance. The essence of this problem lies in leveraging the differences in channel gain among various users within a network. The primary motivation is to amplify the spectral efficiency of the system. A specific strategy to resolve the user pairing/grouping dilemma involves employing a search methodology. When a user pairing/grouping scheme is fixed, the optimal solution can be identified. The next step involves ranking all users in descending order based on the metric ${J}_{m}$. The algorithm in this study for the user pairing/grouping issue is detailed in “Proposed algorithm”. The first step is to delineate the viable solutions for user grouping, considering both the exhaustive and the proposed algorithm. An exhaustive search meticulously examines every data point within the designated search region, ensuring the most accurate match is identified. However, this method demands significant computational resources. This becomes especially challenging for discrete problems where no straightforward solution is apparent. In such cases, it might be necessary to sequentially scrutinize every potential scenario to identify the most suitable solution. Relying solely on the exhaustive search algorithm becomes increasingly challenging as the number of comparisons intensifies quickly.

## Proposed algorithm

This section details the formulation of the proposed Multi-Learning Grey Wolf Optimizer (MLGWO), in addition to the basic concepts of the original GWO algorithm.

### Original GWO algorithm

The Grey Wolf Optimizer (GWO) is an optimization algorithm inspired by the social hierarchy and hunting behaviour of grey wolves in nature^11^. In the wild, grey wolves are known for their strategic hunting techniques, which involve tracking, encircling, and pursuing their prey. This behaviour is mimicked in the GWO algorithm to search for optimal solutions in a given problem space. Grey wolves can be categorized into four main classes based on their social dominance: (i) Alpha ($\alpha$): It is the leader of the pack and makes decisions about hunting, sleeping places, and the time of hunting; (ii) Beta ($\beta$): These wolves assist the alpha in decision-making and take the lead in their absence; (iii) Delta ($\delta$): These wolves obey the alpha and beta but dominate the omegas; (iv) Omega ($\omega$): The lowest ranking wolves.

The mathematical modelling of the GWO algorithm is discussed as follows. Start by initializing a population of grey wolves, where each wolf represents a potential solution in the problem space. Each wolf has a position defined in a multi-dimensional space.

34 $${X}_{i}=\left({x}_{i1},{x}_{i2},\dots ,{x}_{id}\right)$$

where ${X}_{i}$ is the position of the ${i}^{th}$ wolf and $d$ is the dimensionality of the problem. The hunting behaviour of the wolves is modelled using three main steps: encircling the prey, hunting, and attacking. The wolves try to encircle the prey, which is mathematically modelled as follows.

35 $${D}_{{\upalpha },\upbeta ,\updelta }=\left|{C}_{{\upalpha },\upbeta ,\updelta }\times {P}_{{\upalpha },\upbeta ,\updelta }-{X}_{i}\right|$$

36 $${X}_{{\upalpha },\upbeta ,\updelta }={P}_{{\upalpha },\upbeta ,\updelta }-{A}_{{\upalpha },\upbeta ,\updelta }\times {D}_{{\upalpha },\upbeta ,\updelta }$$

where ${C}_{\alpha ,\beta ,\delta }$ are coefficient vectors, ${P}_{\alpha ,\beta ,\delta }$ are the positions of the alpha, beta, and delta wolves, respectively, and ${A}_{\alpha ,\beta ,\delta }$ are random vectors in $[-a, a]$, where $a$ decreases linearly from 2 to 0 over iterations. The position of each wolf is updated based on the positions of the alpha, beta, and delta wolves:

37 $${X}_{new}=\left({X}_{{\upalpha }}+{X}_{\upbeta }+{X}_{\updelta }\right)/3$$

The wolves approach the prey based on a random approach as follows.

38 $$X={X}_{new}+{rand}\times \left({X}_{best}-{X}_{current}\right)$$

where ${X}_{best}$ is the best position found so far, *rand* denotes the random number between 0 and 1, and ${X}_{current}$ is the current position of the wolf. Based on fitness, the wolves are sorted after every iteration. The alpha wolf is the best, the beta wolf is the second best, and the delta wolf is the third best. Up until a stopping condition is satisfied, the algorithm iterates, adjusting the wolves' position in accordance with their hunting behaviour. This could be any other problem-specific constraint, such as a minimal error limit or a maximum number of iterations. The alpha wolf's position indicates the best way to solve the issue once the algorithm has converged. The GWO algorithm looks for the best solution in a given problem area by drawing on the social hierarchy and hunting habits of grey wolves. The algorithm converges when a stopping requirement is satisfied, and the wolf locations are updated in accordance with the best solutions discovered thus far (alpha, beta, and delta).

### Multi-learning Grey Wolf Optimizer

The GWO is a bio-inspired optimization algorithm that simulates the hunting behaviour of grey wolves. While GWO has shown promise in various optimization tasks, it has certain limitations that have motivated researchers to propose improved versions. The primary reasons for improving the performance of the GWO and the problems associated with the original version are as follows. (i) One of the main issues with the original GWO is its tendency to converge prematurely to a local optimum, especially in complex, multi-modal optimization landscapes. Therefore, the algorithm might find a solution quickly, but it might not be the best possible solution; (ii) Over iterations, the wolf agents in GWO can cluster around specific regions of the search space, leading to a lack of diversity. This clustering can further exacerbate the problem of premature convergence as the algorithm becomes less explorative and more exploitative; (iii) Like many optimization algorithms, GWO faces the challenge of balancing exploration and exploitation. The original GWO can sometimes lean towards exploitation, missing out on potentially better solutions in unexplored regions; (iv) For high-dimensional problems, the original GWO might struggle to find optimal solutions within a reasonable computational time; (v) The original GWO uses fixed mathematical equations for updating wolf positions. This lack of adaptability means that the algorithm might not be well-suited for problems that require dynamic adjustments based on the search landscape^90,91^.

To address these challenges, various enhancements, such as integrating competitive learning^92^, Q-learning^93^, and greedy selection^94^, have been proposed in this study. These enhancements aim to increase the diversity of the search agents, guide the search process based on learned experiences, and ensure consistent improvement over iterations, making the algorithm more robust, adaptable, and efficient. Competitive learning can maintain diversity in the population, preventing agents from clustering around specific regions. By making agents compete, competitive learning can ensure a balance between exploring new regions and exploiting known good regions. Q-learning introduces a memory component, allowing the algorithm to learn from past actions and their outcomes. This memory-guided exploration can prevent the algorithm from repeatedly exploring sub-optimal regions. Q-learning allows GWO to adapt its behaviour based on the learned Q-values, making the search process more intelligent. Greedy selection ensures that the algorithm always retains the best solutions, leading to consistent improvement over iterations, and it helps in avoiding sub-optimal decisions made in any single iteration, ensuring that the algorithm is always moving in the right direction.

#### Competitive learning

Competitive learning is a type of neural network learning algorithm where neurons in a layer compete among themselves to be activated. It is often associated with self-organizing maps and vector quantization^92,95^. The fundamental idea is that, given an input, only one neuron is activated, and this neuron is the one whose weight vector is closest to the input vector. In optimization algorithms, competitive learning can be thought of as agents or solutions competing against each other based on their performance or fitness. The competition ensures that only the best agents get selected or updated, leading to a more refined search process. In the proposed MLGWO, competitive learning is introduced as a mechanism where wolves (agents) compete against each other during the optimization process. The following competitive learning strategies are included in the MLGWO. Equation (39) introduces the first competitive learning strategy.

39 $${X}_{new}=X+{\text{rand}}\left(1,{\text{dim}}\right)\times {R}_{3}-{P}_{\text{alpha}}-{\text{rand}}\left(1,{\text{dim}}\right)\times X+\left({P}_{\text{beta}}+{P}_{\text{delta}}\right)$$

Equation (40) introduces the second competitive learning strategy.

40 $${X}_{new}={P}_{\text{alpha}}+{\text{rand}}\left(1,{\text{dim}}\right)\times \left({R}_{2}-{R}_{3}\right)$$

Equation (4) introduces the third competitive learning strategy.

41 $${X}_{new}={P}_{\text{alpha}}+{\text{rand}}\left(1,{\text{dim}}\right)\times \left({R}_{4}-{R}_{5}\right)+{\text{rand}}\left(1,{\text{dim}}\right)\times \left({R}_{6}-{R}_{7}\right)$$

where ${\text{P}}_{\text{alpha}}$ denotes the position of the alpha wolf, ${\text{rand}}$ denotes the uniform random number between 0 and 1, ${\text{d}}{\text{im}}$ denotes the problem dimension, and ${R}_{i}$ are randomly selected solutions from the population. Finally, the update based on competitive learning can be modelled as follows.

42 $${P}_{new}=P+\beta {\sum }_{i=1}^{m}{\lambda }_{i}\left(P-{C}_{i}\right)$$

where $\beta$ is a scaling factor, ${\lambda }_{i}$ are random coefficients in the range [0,1], determining the influence of each competitor, and $P$ denotes the wolf's position. A set of competitor wolves with positions ${C}_{i}$, where $i = 1, 2, \dots , m$ and $m$ is the number of competitors. Equation (42) represents the wolf's position being updated based on the differences between its position and the positions of its competitors. The random coefficients ${\lambda }_{i}$ ensure variability in the updates, promoting exploration. For each wolf in the population, a subset of other wolves is randomly selected as competitors. This subset does not include the current wolf. The current wolf's position is updated based on its interaction with the selected competitors. This update can be based on various strategies, such as (i) using the difference between the positions of two randomly selected competitors and (ii) combining the differences between multiple pairs of competitors. As wolves compete, they are forced to explore different regions of the search space, ensuring diversity. This is crucial in preventing premature convergence to local optima. Competitive learning strikes a balance between exploration and exploitation. While the competition ensures that wolves are constantly trying to outdo each other, the best-performing wolves guide the search towards promising regions. By making wolves compete against each other, competitive learning ensures that the search space is explored more thoroughly. In standard GWO, wolves can sometimes cluster around specific regions, leading to stagnation. Competitive learning disrupts this clustering by constantly pushing wolves to outperform their competitors. The competitive mechanism allows the algorithm to adapt its behaviour based on the current state of the search. If a wolf finds itself in a less promising region, the competition will likely push it towards a better region. Competitive learning makes the algorithm more robust against getting trapped in local optima. The constant competition ensures that even if some wolves converge to a local optimum, others continue to explore and potentially find better solutions. In summary, competitive learning introduces a dynamic, adaptive competition mechanism into GWO, ensuring a more thorough exploration of the search space, maintaining diversity, and enhancing the algorithm's robustness and adaptability. The features of combining GWO and competitive learning are as follows. (i) The variability in updates ensures that different regions of the search space are explored; (ii) By being influenced by multiple competitors, wolves are less likely to cluster around specific regions, preventing stagnation; and (iii) While the competition promotes exploration, the influence of the best-performing competitors ensures that the search is also guided towards promising regions. The pseudocode for competitive learning is provided in *Algorithm 1*.

**Algorithm 1:**
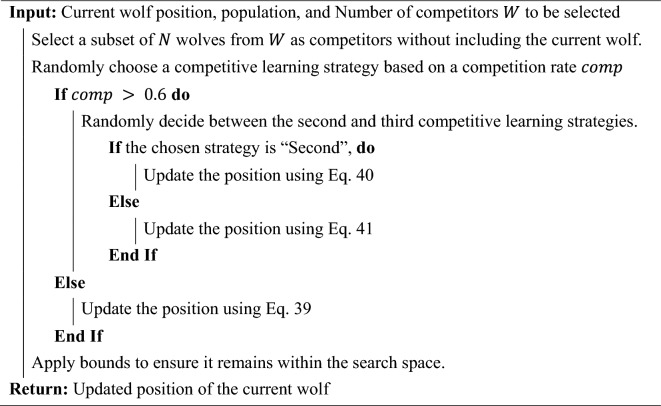
Pseudocode for the competitive learning.

#### Q-learning integration

Q-learning is a model-free reinforcement learning algorithm used to find the optimal action-selection policy for a given finite Markov decision process^96,97^. It works by learning an action-value function and using it to choose the most valuable action at each state. Each agent maintains a Q-table, allowing it to make informed decisions on its movements in the search space. This memory-guided exploration ensures that the algorithm learns from past actions, preventing it from repeatedly exploring sub-optimal regions and enhancing its adaptability. In the proposed MLGWO, Q-learning is integrated to guide the search of the agents in the optimization landscape based on the learned Q-values. The Q-values represent the expected future rewards for taking an action in a particular state. The Q-learning update rule is as follows.

43 $$Q\left({s}_{t},{a}_{t}\right)=Q\left({s}_{t},{a}_{t}\right)+{\upalpha }\times \left({r}_{t+1}+\upgamma \times \underset{a}{\text{max}}Q\left({s}_{t+1},a\right)-Q\left({s}_{t},{a}_{t}\right)\right)$$

where ${s}_{t}$ is the current state, ${a}_{t}$ is the action taken, ${r}_{t+1}$ denotes the reward received after taking action ${a}_{t}$ in state ${s}_{t}$. ${\upalpha }$ is the learning rate, and $\upgamma$ is the discount factor. In this study, states are the current positions of the agents, and actions are the decisions made by the agents to move in the search space. Q-learning introduces a memory component, allowing the algorithm to learn from past actions and their outcomes. This memory-guided exploration can prevent the algorithm from repeatedly exploring sub-optimal regions. Q-learning allows GWO to adapt its behaviour based on the learned Q-values, making the search process more intelligent. The following steps are followed in this study.

- Initialize the Q-table with zeros. The Q-table table has states as rows and actions as columns. In this study, states can be the positions of the wolves, and actions are the decisions made by the wolves to move in the search space.
- At each iteration, for each agent, select an action based on the Q-values, and it can be done using an $\varepsilon$-greedy strategy where with probability $\varepsilon$, a random action is chosen, and with probability $1-\varepsilon$, the action with the highest Q-value for the current state is chosen.
- After taking action and observing the new state and reward, update the Q-values using the Q-learning update rule.
- After several iterations, the Q-values converge, and the policy (optimal action for each state) can be extracted from the Q-table.

The pseudocode for the Q-learning is provided in Algorithm 2.

**Algorithm 2:**
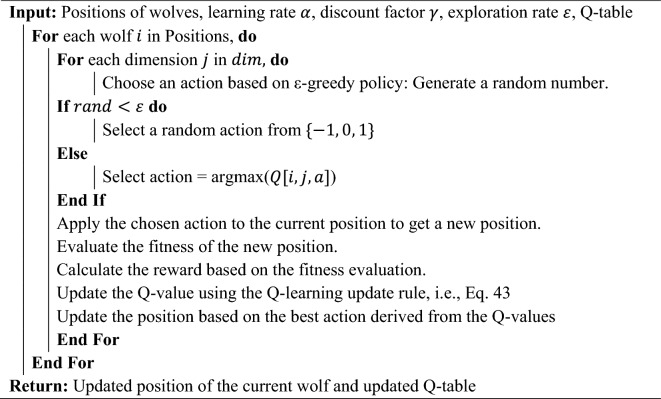
Pseudocode for the Q-learning.

Q-learning allows the GWO to build a knowledge base from past iterations. Each agent (grey wolf) records the outcomes of its actions in a Q-table, where the state-action pairs are associated with a value representing the quality (Q-value) of taking a specific action in a given state. The Q-value is updated iteratively based on the rewards received from the environment (i.e., the improvement in solution quality). By referencing the Q-table, agents can make informed decisions about which actions are likely to yield the best results based on historical performance. This guidance helps in avoiding previously explored suboptimal regions and directs the search towards more promising areas of the solution space. Consequently, this enhances the efficiency of the search process, ensuring that computational resources are utilized effectively. Q-learning inherently balances exploration and exploitation through its action-selection strategy, often implemented using an epsilon-greedy approach. Initially, agents are encouraged to explore various actions to gather comprehensive knowledge about the solution space. As the optimization progresses and the Q-table becomes more accurate, the agents increasingly exploit known high-quality actions to refine their solutions. The Q-values are continuously updated based on new experiences, allowing the optimizer to adapt dynamically to the evolving search landscape. This adaptability ensures that the search process remains relevant and effective even as the optimizer discovers new high-potential regions. By learning from past actions and adjusting future actions accordingly, Q-learning enhances the overall robustness and reliability of the optimization process.

#### Greedy selection

Greedy selection is about making the locally optimal choice at each stage with the hope of finding a global optimum. In this study, it is about selecting the best solutions between the standard GWO and the MLGWO at each iteration. Greedy selection ensures that the algorithm always retains the best solutions found so far, leading to consistent improvement over iterations. By always choosing the best available solutions, the algorithm avoids getting stuck with sub-optimal decisions made in any single iteration. By consistently keeping the best solutions, the algorithm can converge faster to the optimal or near-optimal solution.

At every decision point, choose the option that seems best at the moment. This means that the algorithm makes a series of locally optimal choices. Greedy algorithms do not consider the broader implications of a choice; they pick the best option available right then. This can be both an advantage (due to its simplicity and speed) and a disadvantage (as it might not lead to the global optimum). Greedy algorithms are used in a wide range of applications, from network routing task scheduling to some classic optimization problems like the Knapsack problem, Huffman coding, and the coin change problem. After each iteration or update, evaluate the fitness of all solutions or agents. Compare the new solutions with the previous ones. Retaining the solution that is better between the new and the old one is the "greedy" step, as the algorithm immediately accepts any improvement without considering any long-term implications. By always choosing the best solution available at each step, the algorithm can quickly move towards better solutions. Greedy selection is straightforward to implement and understand. It will not require complex decision-making processes. By continuously opting for better solutions, the algorithm can avoid getting stuck or stagnating at sub-optimal solutions for extended periods.

The primary limitation of greedy selection is that it can lead the algorithm to converge to local optima. Since it doesn't consider the broader landscape of the solution space, it might miss better solutions that would require initially moving to a worse position. Greedy algorithms can be overly exploitative, focusing too much on refining the current best solutions and not enough on exploring new regions of the solution space. In the proposed MLGWO, greedy selection ensures that after each iteration, the best solutions are retained. This helps in consistently moving towards optimal or near-optimal solutions and provides a mechanism to ensure that the algorithm doesn't regress in its search for the best solution.

**Fig. 3:**
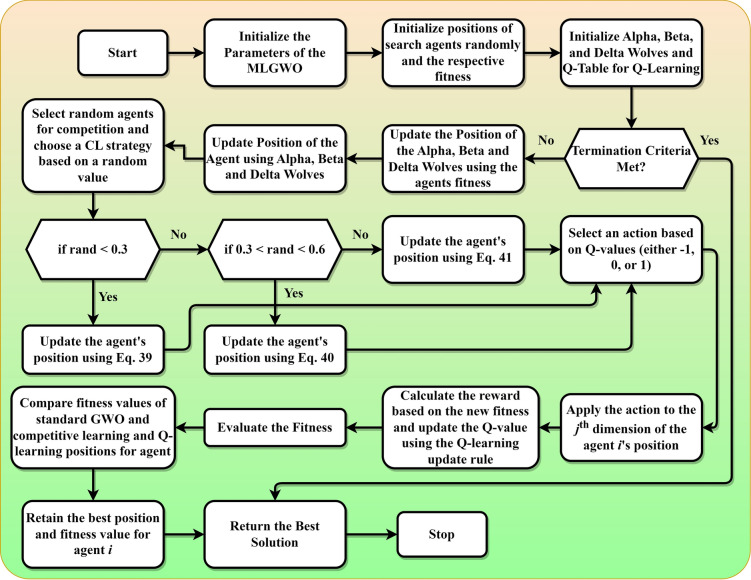
Flowchart of the proposed MLGWO.

Finally, various enhancements, such as integrating competitive learning, Q-learning, and greedy selection, have been proposed in this study. These enhancements aim to increase the diversity of the search agents, guide the search process based on learned experiences, and ensure consistent improvement over iterations, making the algorithm more robust, adaptable, and efficient. By combining these mechanisms, the GWO can achieve a more balanced and efficient search, potentially leading to faster convergence, better solution quality, and a higher likelihood of escaping local optima. The pseudocode of the proposed MLGWO is presented in *Algorithm 3*. The flowchart of the MLGWO is shown in Fig. 3.

**Algorithm 3:**
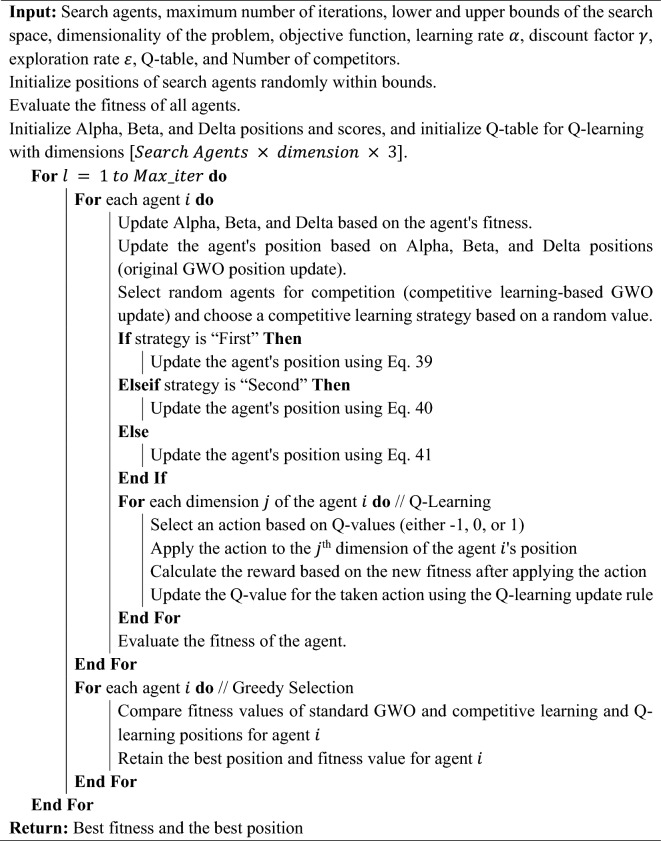
Pseudocode of the proposed MLGWO algorithm.

### System complexity

The MLGWO integrates Competitive Learning, Q-learning, and Greedy Selection, which streamline the optimization process. This integration reduces redundant calculations by focusing on high-potential solutions and dynamically adapting search strategies. In contrast, the standard GWO lacks these adaptive mechanisms, leading to higher iterations and increased complexity. Golden Jackal Algorithm (GJA) involves complex adaptive mechanisms for balancing exploration and exploitation, contributing to higher system complexity. PSO requires extensive parameter tuning and velocity updates, adding to the computational burden. The Slime Mould Algorithm (SMA) is known for its adaptive mechanisms based on slime mould behaviour and also suffers from increased complexity due to its iterative nature and complex biological inspirations.

The position matrix stores the positions of all search agents. Its size is $N\times dim$, so its space complexity is $O\left({\text{N}}\times {\text{dim}}\right)$. The Q-values for Q-learning are stored in a 3D matrix of size $N\times dim\times 3$. Its space complexity is $O\left({\text{N}}\times {\text{dim}}\right)$. The overall space complexity is dominated by the ‘Positions’ matrix and the Q-table, making it $O\left({\text{N}}\times {\text{dim}}\right)$.

Initializing the positions and evaluating their fitness values takes $O\left({\text{N}}\times {\text{dim}}\right)$. The main loop runs for ‘$Max\_iter$’ iterations. Within each iteration: (i) Updating the positions of the agents based on the alpha, beta, and delta positions involves operations proportional to ${\text{N}}\times {\text{dim}}$; (ii) The competitive learning-based update also involves operations proportional to ${\text{N}}\times {\text{dim}}$; (iii) The Q-learning update, including action selection and Q-value update, involves operations proportional to ${\text{N}}\times {\text{dim}}$. Given that these operations are sequential and not nested, the time complexity within the main loop is $O\left({\text{N}}\times {\text{dim}}\right)$. Since the main loop runs for the maximum number of iterations, the overall time complexity is $O\left({\text{Max}}\text{\_}{\text{iter}}\times {\text{N}}\times {\text{dim}}\right)$.

The proposed MLGWO offers significant advantages over standard GWO, GJA, PSO, and SMA despite sharing a similar theoretical complexity. MLGWO's integration of Competitive Learning, Q-learning, and Greedy Selection ensures faster convergence and reduced computational time by requiring fewer iterations to achieve high-quality solutions. This efficiency contrasts with the standard GWO's higher iteration count, GJA's complexity from adaptive mechanisms, PSO's increased demands in high-dimensional problems, and SMA's computational overhead from intricate biological-inspired updates.

## Results and discussions

### Case study descriptions

The section evaluates the performance of a NOMA uplink scenario. Precisely, it looks at how well a proposed user-grouping algorithm and power control approach works. MATLAB, a high-level language and interactive environment used for numerical computation, visualization, and programming, is used for the simulation process. The parameters used for the simulation are taken from existing literature and are listed in Table 1. The channel and position of the user are dispersed at random. This means that in the simulation, users can be anywhere and, on any channel, representing real-world unpredictability. The distance (or range) between the BS and the user is uniformly distributed, and it means that any distance between the user and the BS is equally likely. The channel response follows a Gaussian distribution. In simpler terms, most users have a channel response close to the average, with fewer users having exceptionally good or bad channel responses. This section aims to compare the effectiveness of the proposed algorithm established for (i) power control, (ii) user grouping, and (ii) decoding order in NOMA uplink systems. The proposed algorithm's performance is compared with four other algorithms, such as original GWO, SMA, PSO, and GJA. All algorithms, including the proposed MLGWO, are nature-inspired optimization techniques. They mimic certain behaviours observed in nature to find optimal or near-optimal solutions to problems. Each has its unique characteristics and mechanisms, making them suitable for different types of optimization challenges. The summary of all algorithms, except GWO and the proposed MLGWO, is presented as follows.

**Table 1:** Simulation parameters of the uplink NOMA transmission.

Parameter	Value
Number of PRB/Group $K$	3
Number of users $N$	10
Number of cells $C$	1
Transmission power to noise ratio $\gamma$	30 dB
Minimum transmission rate requirement ${r}_{n}$	1.1 bits/s/Hz

SMA: A population-based optimization technique that mimics the behaviour of slime mould is called the SMA. The SMA replicates the positive and negative feedback of the slime mould propagation wave using a mathematical model. The SMA mimics the ways in which slime mould moves and forages. To guarantee that they receive the highest possible concentration of substances, slime moulds can form a stronger path where food concentration is higher. It is used in network design problems, pathfinding, and other optimization tasks.

PSO: The PSO iteratively refines a potential solution based on a predetermined quality criterion and is modelled after the cooperative behaviour of real animals, such as fish or birds, that move towards a shared objective. In 1995, Russell Eberhart and James Kennedy originally introduced PSO. It is used for a variety of optimization and search problems. PSO moves a group of people known as particles through an area in stages, evaluates the objective function of each particle at each step, and multiplies the current velocity by an inertia variable. Both the social and cognitive coefficients are used. The theory states that the possibility of creating new particles is maximized in the right direction. PSO is versatile and has been applied to a wide range of optimization problems, including function optimization, neural network training, and system design.

GJA: The GJA is inspired by the social and hunting behaviour of golden jackals. Similar to the GWO, the golden jackal algorithm mimics the hunting behaviour of jackals. Jackals can hunt both individually and in small groups. The algorithm captures this behaviour to search and converge to an optimal solution. The GJA can be applied to various optimization problems, similar to the other nature-inspired algorithms.

The specific control parameters for these algorithms are provided in Table 2. The population size refers to the number of potential solutions that the algorithms start with. In this study, the population size is 30. The maximum number of iterations is 500 iterations. Each algorithm is run 30 times to ensure that the results are consistent. By running the algorithm multiple times, the users get a better sense of its average performance.

**Table 2:** Control parameters of the selected algorithms.

Algorithms	Parameters	Value
GWO	$a$	Linearly varying from 2 to 0
MLGWO	$a$	Linearly varying from 2 to 0
	Learning rate	0.6
	Discount factor	0.8
	Exploration rate	0.1
	Number of competitors	5
PSO	Inertial weight	0.9
	Coefficient $C1$ and $C2$	2
SMA	$z$	0.03
GJA	$a$	1.5 (linearly decreased over iterations)

### User grouping strategies

This sub-section presents the simulation results for various user grouping strategies aimed at optimizing spectral efficiency and achievable sum rate using the proposed MLGWO in 5G uplink communication systems. Five strategies were evaluated: random, channel-based, distance-based, equal-power, and mixed grouping. It is also aimed to demonstrate the superior performance of the proposed MLGWO algorithm and other NOMA and OMA strategies in terms of spectral efficiency.

Random grouping serves as a baseline, where users are assigned to groups without considering channel conditions, distances, or power levels. This strategy provides a reference point for evaluating the effectiveness of more sophisticated grouping methods. As expected, the performance of random grouping is suboptimal, showing significant variability due to the lack of optimization. The spectral efficiency and sum rates are the lowest among all strategies, highlighting the importance of informed user grouping. Channel-based grouping prioritizes users with stronger channel gains, assigning them to the same group. This strategy significantly enhances performance metrics, as evidenced by higher spectral efficiency and achievable sum rates. The rationale behind this improvement is that users with stronger channels can better utilize the available power, leading to higher data rates. This strategy is particularly effective in environments with substantial channel gain variations, where exploiting these gains results in superior performance. Distance-based grouping focuses on user proximity to the base station, grouping users with similar distances together. This strategy aims to minimize path loss and signal degradation. The performance improvements are moderate compared to the channel-based approach. While it provides better spectral efficiency and sum rates than random grouping, it does not fully exploit channel conditions. However, in scenarios where user distances significantly impact signal strength and interference, this strategy offers noticeable benefits. The equal-power grouping strategy seeks to balance power distribution among groups, ensuring each group receives an equitable share of the available power. This approach results in some performance gains over random grouping, but it falls short of the channel-based and mixed strategies. The main limitation is that it does not consider channel gains or user distances, leading to suboptimal use of the available resources. Consequently, the spectral efficiency and sum rates are lower than those achieved by strategies that leverage more comprehensive information. Mixed grouping combines the strengths of channel-based and distance-based strategies. By considering both channel gains and user proximity, this approach adapts to varying conditions more effectively. The mixed strategy consistently yields the best performance, with the highest spectral efficiency and sum rates among all strategies. This is because it can dynamically adjust to different scenarios, exploiting strong channels while also minimizing path loss and interference. The synergy of channel and distance information results in balanced and optimal user grouping.

**Fig. 4:**
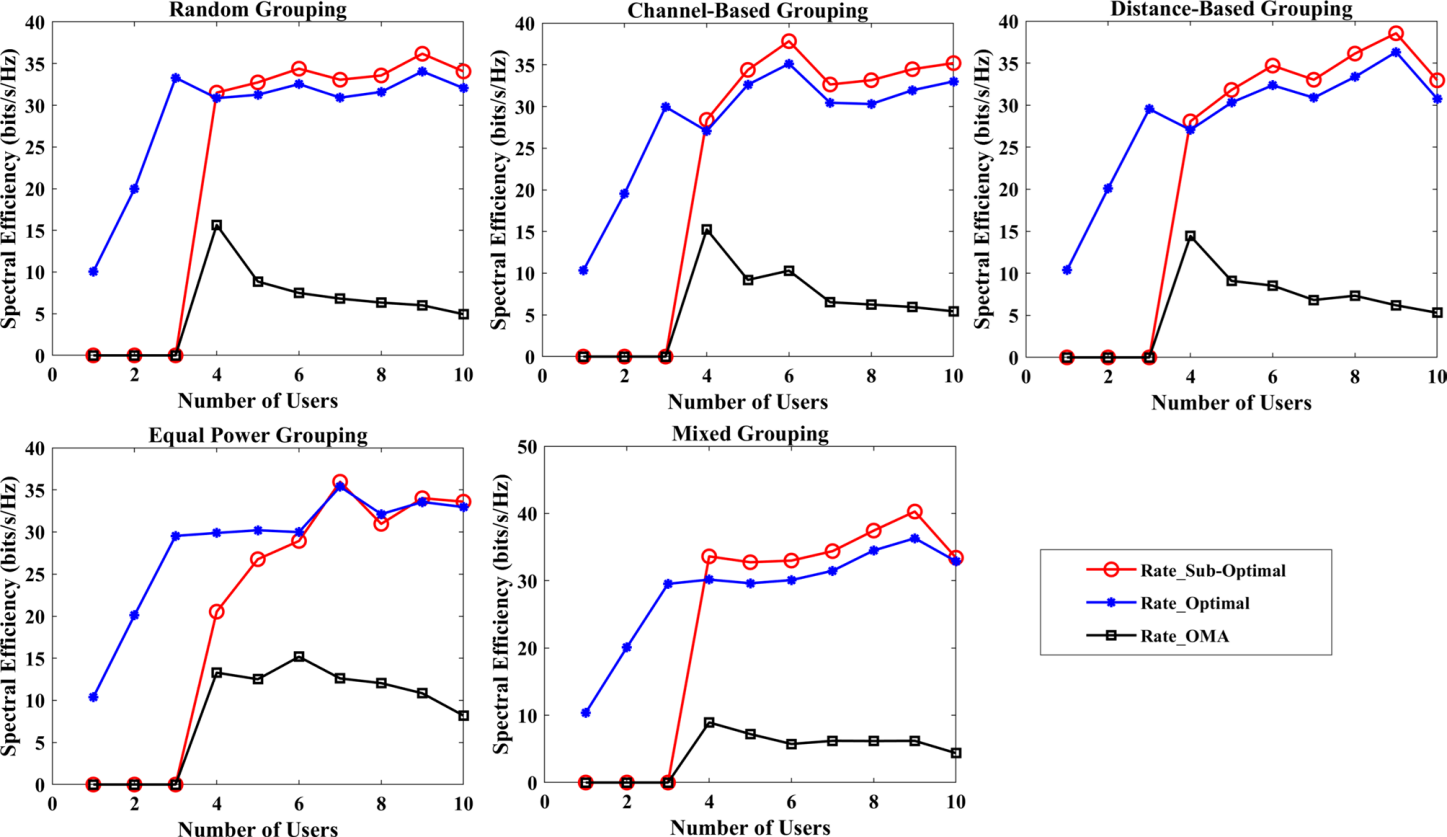
Spectral efficiency achieved by MLGWO for different user grouping strategies.

In channel-based grouping, the MLGWO algorithm efficiently identifies users with strong channels and groups them to maximize spectral efficiency. By dynamically adjusting the positions of search agents (representing user groupings), MLGWO ensures that the grouping strategy quickly converges to an optimal or near-optimal solution. In distance-based grouping, the MLGWO helps minimize path loss by effectively grouping users based on their distances from the base station. The algorithm iteratively refines the user groupings to ensure that users with similar distances are grouped together, enhancing signal strength and reducing interference. In the mixed grouping, the MLGWO algorithm's adaptive nature allows it to balance the influence of channel gains and user proximity. By iteratively adjusting the groupings based on real-time feedback from the objective function, MLGWO ensures that the mixed strategy can dynamically adapt to varying conditions, providing robust performance across different scenarios. The spectral efficiency of different user grouping strategies is presented in Fig. 4.

Since random grouping does not rely on optimization, the impact of MLGWO is minimal. The performance remains relatively low, serving as a baseline for comparison. In channel-based grouping, the proposed MLGWO optimizes the selection of users with strong channel gains, leading to higher spectral efficiency and sum rates. The algorithm ensures that the best possible groupings are identified quickly and effectively, leveraging the channel conditions to maximize performance. In distance-based grouping, the proposed MLGWO improves the grouping of users based on their proximity to the base station. While the impact is less pronounced than in channel-based grouping, the algorithm still enhances performance by minimizing path loss and improving signal strength. In equal power grouping, the MLGWO helps balance power distribution among groups more effectively than a non-optimized approach. However, the lack of consideration for channel gains and distances limits the overall impact of MLGWO compared to other strategies. The proposed MLGWO has the most significant impact on mixed grouping. By balancing channel gains and user distances, the algorithm ensures optimal groupings that adapt to varying conditions. The iterative refinement process of MLGWO leads to the highest spectral efficiency and sum rates among all strategies.

From the results, it is observed that channel-based and mixed strategies consistently outperform others, demonstrating significant improvements in spectral efficiency. Their ability to improve channel gains and user proximity results in superior performance. The channel-based and mixed strategies converge faster, indicating their efficiency in optimizing user grouping. The distance-based and equal-power strategies offer moderate improvements but do not fully improve available information for optimal performance. Random grouping serves as a baseline, illustrating the need for informed user grouping to achieve higher performance metrics.

### Experimental results

**Fig. 5:**
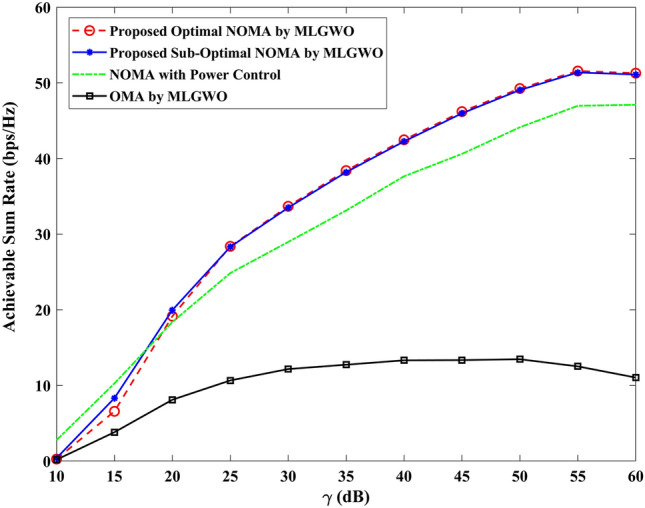
Achievable sum rate in bps/Hz by the proposed MLGWO.

This study aimed to demonstrate the superior performance of the proposed MLGWO algorithm over other existing algorithms and other NOMA and OMA strategies in terms of spectral efficiency. To achieve this, the study conducted a series of simulations and plotted the results to represent the performance metrics visually. The bandwidth settings in the simulations are chosen to reflect typical values used in 5G and beyond networks. Bandwidths of 10 MHz, 20 MHz, and higher are commonly employed to simulate realistic communication scenarios. These settings are in line with the bandwidth allocations specified for 5G networks by regulatory bodies such as the 3rd generation partnership project^98,99^. In the study, a comparison is made between the achievable sum rate of two communication approaches: NOMA and OMA. This comparison is visually represented in Fig. 5. The SNR from 10 to 60 dB used in the simulations cover a broad spectrum of realistic channel conditions. This range allows us to assess the system performance from moderate to high SNR scenarios, providing a comprehensive analysis of the achievable sum rate in NOMA uplink systems. The choice of this range is supported by established literature^100–103^, which frequently employs similar SNR ranges to evaluate the performance of communication systems. Using SNR from 10 to 60 dB enables us to evaluate the performance of the proposed MLGWO across a wide range of operational conditions. This comprehensive evaluation is crucial for understanding how the algorithm performs under varying signal quality, which directly impacts the achievable sum rate. By covering both moderate and high SNR scenarios, it is ensured that the simulation results provide a realistic and thorough assessment of the algorithm's effectiveness. The chosen SNR is reflective of practical scenarios encountered in real-world wireless communication systems. Higher SNR values (up to 60 dB) are particularly relevant for assessing performance in environments with strong signal strength and low noise, which are common in advanced 5G and beyond networks. This range allows us to demonstrate the robustness and scalability of our proposed algorithm under optimal conditions, as well as its effectiveness in less favourable conditions. By using these SNR values in the simulations, it is ensured that the results are both realistic and comparable with existing literature, providing a robust foundation for evaluating the performance of the proposed MLGWO in optimizing spectral efficiency in NOMA uplink systems.

The spectral-efficiency metric is crucial in communication systems as it measures how efficiently the spectral bandwidth is utilized. A higher spectral efficiency indicates that more data can be transmitted over a given bandwidth, making the communication system more efficient. From the data presented, it is evident that the NOMA approach outperforms the OMA approach in terms of spectral efficiency. Specifically, the spectral efficiency achieved by the NOMA scheme is significantly higher than that of the OMA scheme. This superiority of NOMA over OMA in spectral efficiency is a notable finding. The study also introduces a sub-optimal approach for NOMA. Interestingly, the spectral efficiency of this sub-optimal approach is very close to what would be considered the optimal value. This means that even though the approach might not be the absolute best possible, it is very close to achieving the highest spectral efficiency. The proposed MLGWO plays a pivotal role in this study. The MLGWO is designed to find near-optimal solutions. One of the standout features of the MLGWO is that it achieves this near-optimal performance without requiring extensive computational resources and makes the proposed MLGWO both efficient and effective. As the number of users in the system increases, the computational demands of certain algorithms also rise. The exhaustive search algorithm, for instance, sees a significant increase in its computational cost as more users are added. In contrast, the MLGWO maintains a relatively low computational complexity, making it a more scalable and practical choice for systems with a large number of users. For NOMA uplink systems, a benchmark is set using the power control approach detailed in the study by^35^. The spectral efficiency values achieved in their study are close to the optimal value, making it a valuable reference point. However, it is crucial to note a limitation in^35^: it is designed to work effectively only for systems with two user-pairings. Given this limitation, the proposed scheme in the current study offers a significant advantage. It is adept at efficiently grouping multiple users, not just two. This capability ensures that the proposed scheme is versatile and can handle scenarios with more than just a pair of users, making it a more comprehensive solution for NOMA uplink systems. Therefore, the study provides a thorough comparison of NOMA and OMA approaches, highlighting the superior spectral efficiency of NOMA. The proposed sub-optimal approach and the MLGWO further enhance the efficiency and effectiveness of the NOMA system, especially when dealing with multiple users.

**Fig. 6:**
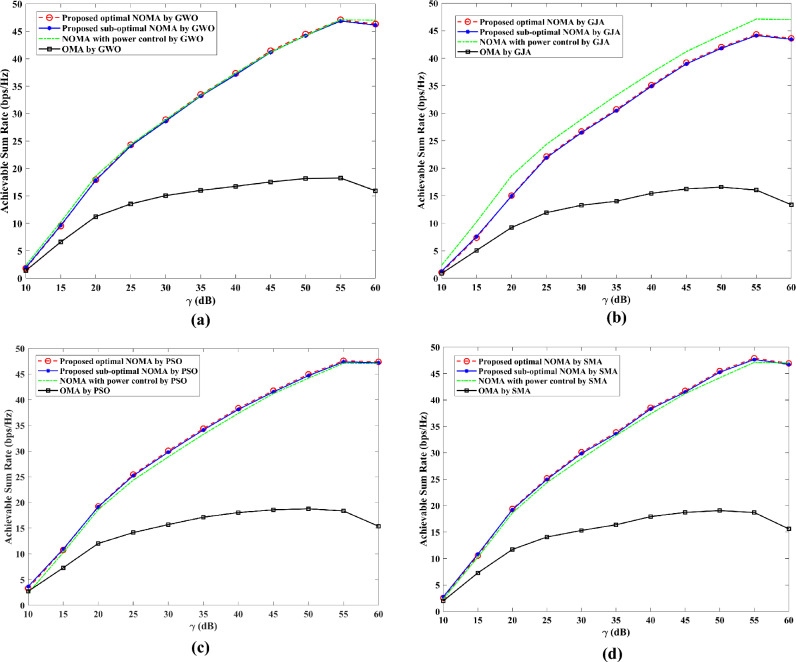
Achievable sum rate in bps/Hz by: (**a**) GWO, (**b**) GJA, (**c**) PSO, (**d**) SMA.

In addition, the study has also presented an achievable sum rate, i.e., spectral efficiency obtained from various competitive algorithms. Figure 6a–d present a comprehensive evaluation of the performance of four distinct algorithms: the GWO, the GJA, the PSO, and the SMA. The primary metric used to assess the performance of these algorithms is spectral efficiency. An interesting observation from the study is the closeness in performance between the optimal and sub-optimal solutions. In many scenarios, the difference between an optimal solution and a sub-optimal solution can be significant. However, in this context, the spectral efficiency of both solutions is remarkably close to each other. This suggests that even if the system does not achieve the absolute best solution, the performance drop is not significant, and the system still operates near its peak efficiency.

The study by^35^ provides a power control scheme specifically designed for the NOMA uplink system. This scheme is employed as a benchmark or reference point in the current study. Benchmarks are essential as they offer a standard against which new or alternative solutions can be compared. Upon analysis, a noteworthy observation emerges that the spectral efficiency achieved by GWO, PSO, and SMA surpasses the results of both the power control scheme from^35^ and the OMA scheme. However, the performance of GJA is worse compared to all algorithms and the benchmark results but better than the OMA scheme. This is a significant finding as it underscores the effectiveness and superiority of the evaluated algorithms in optimizing spectral efficiency. Therefore, Fig. 4 offers a deep dive into the performance of four nature-inspired algorithms in the realm of spectral efficiency. The results highlight the prowess of the NOMA scheme over OMA and emphasize the near-par performance of optimal and sub-optimal solutions. Furthermore, the assessed algorithms not only meet but exceed the benchmark set by^35^ (except GJA), showcasing their potential to enhance communication systems.

**Fig. 7:**
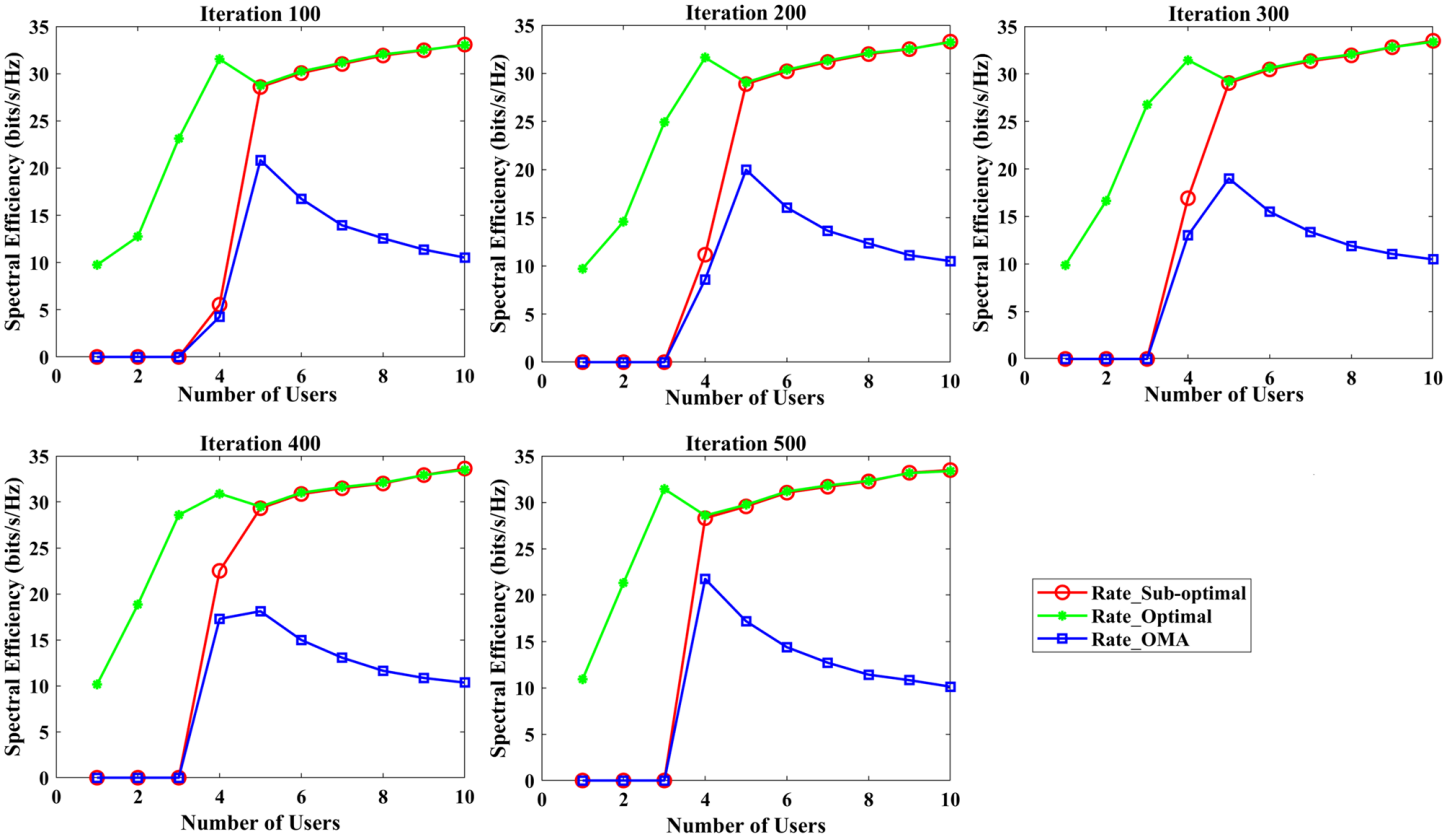
Performance of MLGWO over different iterations for both NOMA and OMA.

**Fig. 8:**
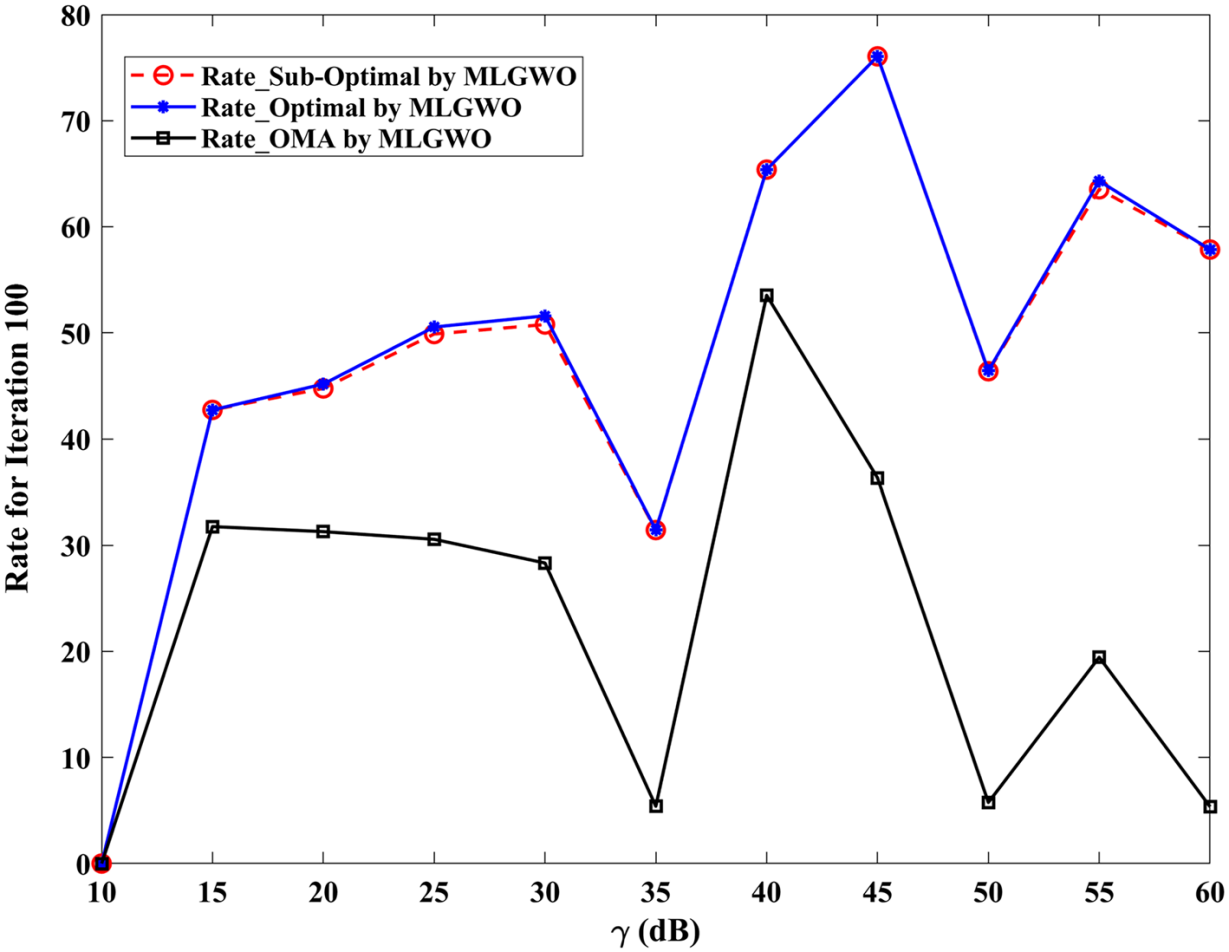
Rates obtained for 100 iterations by MLGWO for the NOMA and OMA.

**Fig. 9:**
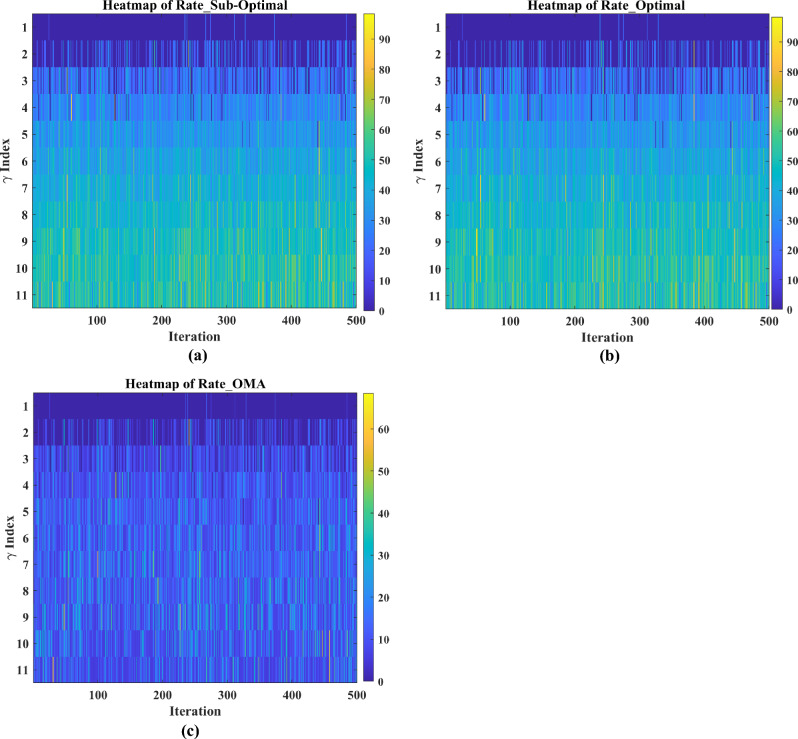
Heatmaps generated obtained for 500 iterations by MLGWO: (**a**) sub-optimal NOMA, (**b**) optimal NOMA, (c) OMA.

Figure 7 shows the spectral efficiency versus the number of users for different iterations. In this plot, the study compared the spectral efficiency against the number of users for different iterations, specifically for 100, 200, 300, 400, and 500 iterations. The proposed algorithm consistently achieved higher spectral efficiency across all user counts when compared to other algorithms. As the number of iterations increased, the spectral efficiency of the MLGWO algorithm showed a more pronounced improvement, indicating the algorithm's ability to optimize better over time. Figure 8 shows the rates for specific iterations, i.e., 100. By focusing on a specific iteration, it is possible to delve deeper into the performance distinctions. Even at specific iterations, the MLGWO consistently outperformed others, further solidifying its superiority. Figure 9 shows the heatmaps of rates, and it provides a visual representation of the rate distributions over iterations and power levels. The colour intensity in the heatmap corresponding to the MLGWO was consistently higher, indicating better rate achievements across the board.

**Fig. 10:**
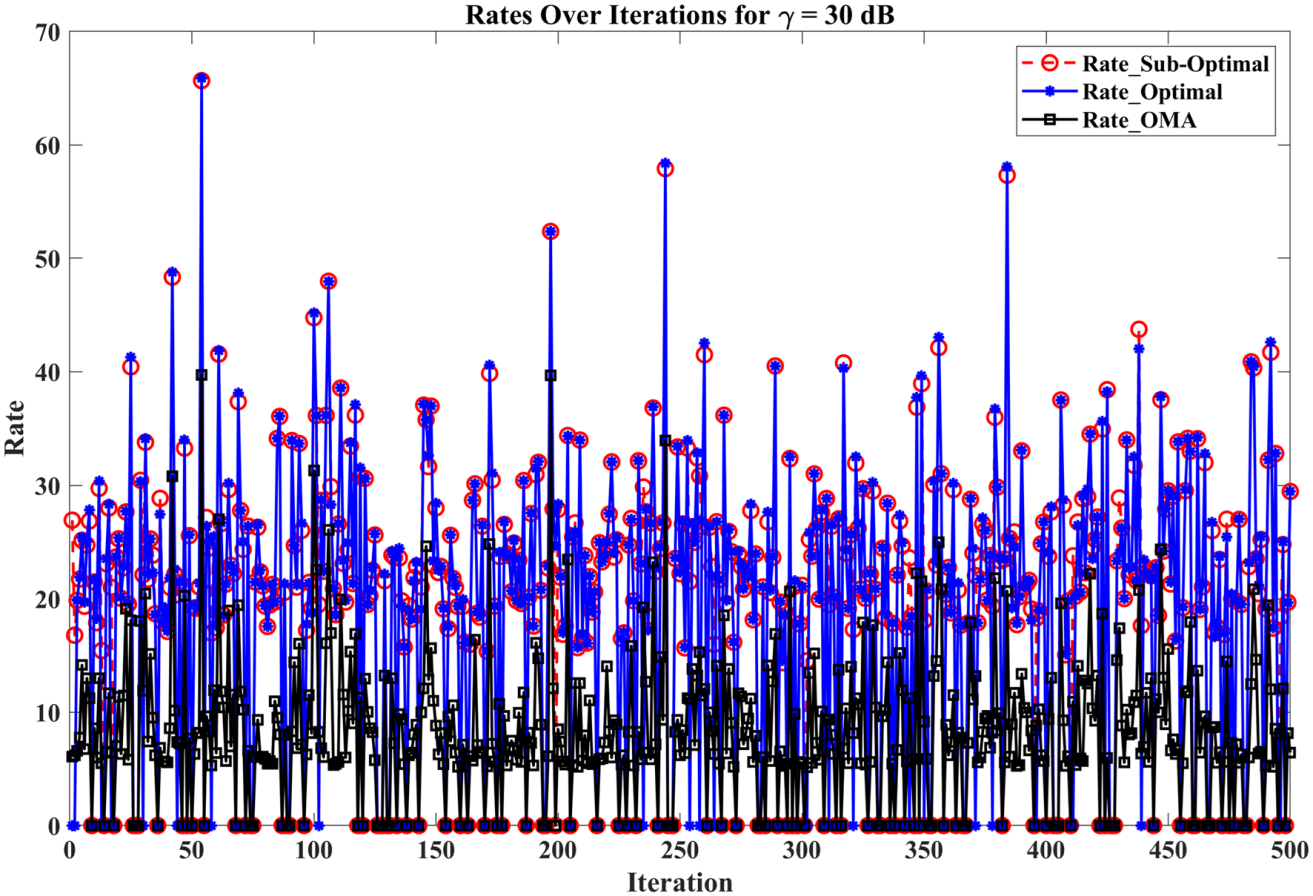
Rates at $\upgamma$=30 dB were obtained for 500 iterations by MLGWO for sub-optimal NOMA, optimal NOMA and OMA.

**Fig. 11:**
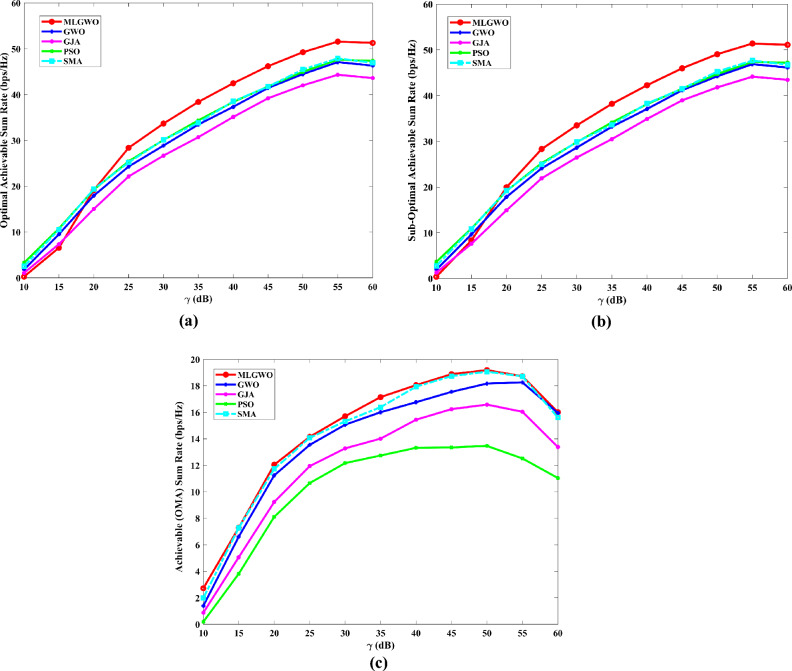
Performance comparison among all algorithms: (**a**) Optimal NOMA, (**b**) Sub-optimal NOMA, (**c**) OMA.

Figure 10 shows the rates over iterations for specific power levels, and this plot provides a detailed view of rate variations over iterations for a chosen power level. The proposed algorithm's curve was consistent, showcasing its ability to maintain superior performance across iterations. Figure 11a–c present a meticulous comparison of various algorithms and approaches in the context of spectral efficiency. Upon analyzing Fig. 11a–c, it becomes evident that the performance of the proposed optimal, sub-optimal, and OMA approaches, when evaluated using the algorithms GWO, GJA, PSO, and SMA, are remarkably close to each other. This means that there is no significant difference in spectral efficiency among these approaches when assessed using the mentioned algorithms. Such a close performance indicates that each approach, regardless of being optimal, sub-optimal, or traditional (OMA), can achieve near-similar results when optimized using these algorithms. However, the spectral efficiency achieved by the proposed MLGWO is remarkably better than all the selected algorithms and traditional approaches. The study further delves into the performance of these algorithms at higher values of the parameter, specifically when this value exceeds 20 dB. In such scenarios, a standout observation is made: the optimal approach, when evaluated using the MLGWO, exhibits superior spectral efficiency compared to all other algorithms.

This suggests that at higher values of $\gamma$, the MLGWO algorithm, when applied to the optimal approach, is the most efficient in maximizing spectral efficiency. Figure 11 offers a detailed comparison of various communication approaches using different optimization algorithms. While the performance of these approaches is closely matched across the algorithms, the MLGWO shines in scenarios with higher values of $\gamma$, showcasing its potential to enhance spectral efficiency in such conditions.

**Fig. 12:**
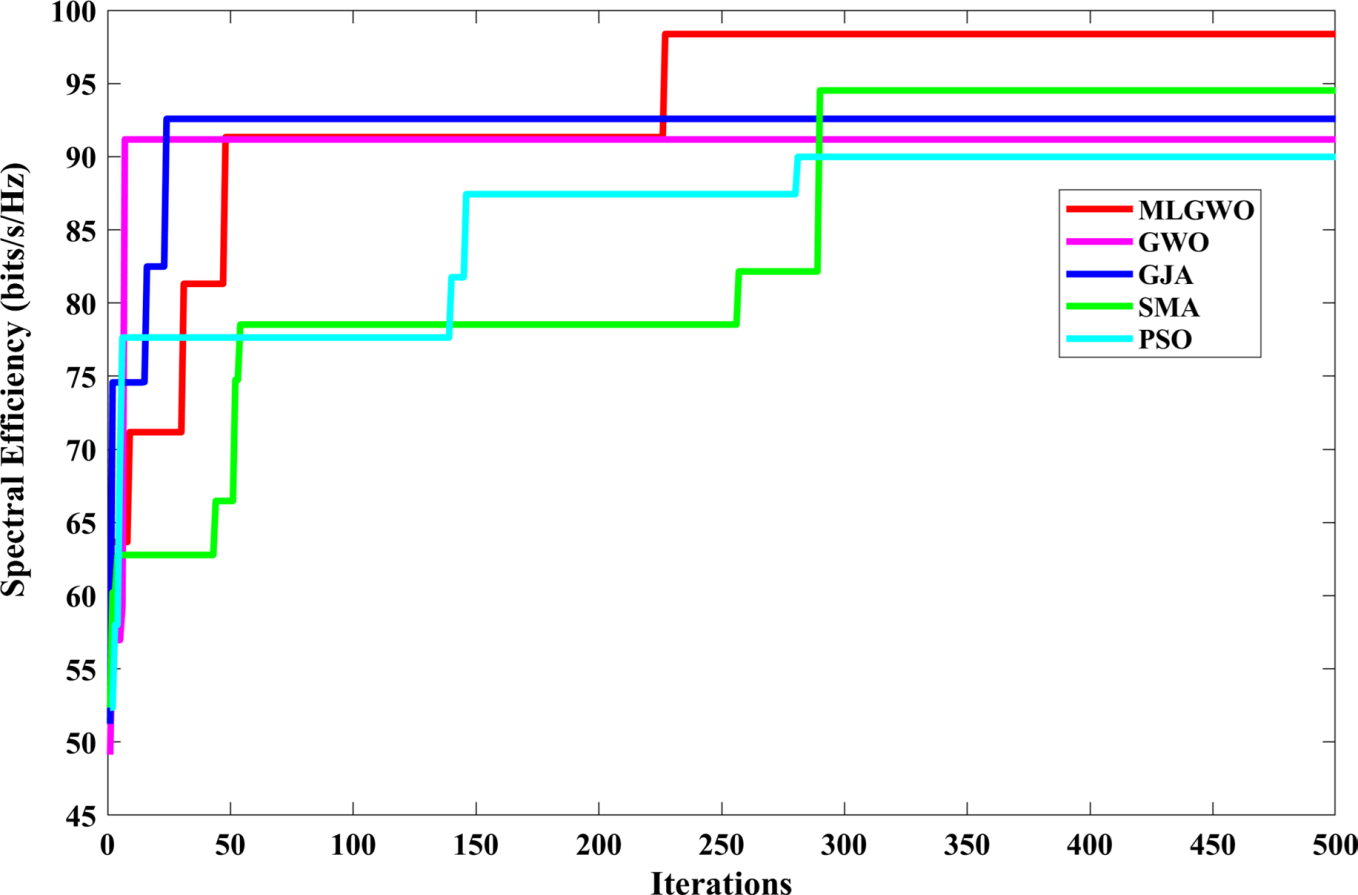
Convergence curves obtained by all selected algorithms.

Figure 12 provides an in-depth analysis of the convergence rates of all algorithms applied to the NOMA uplink system. Upon analyzing Fig. 12, it becomes evident that the MLGWO, GWO, GJA, PSO, and SMA algorithms all exhibit similar convergence rates when applied to the NOMA uplink system. This means that these algorithms, when tasked with finding the optimal or near-optimal solution for the system, take roughly the same number of iterations. However, a notable exception is observed with the MLGWO. The MLGWO takes longer to converge, specifically requiring over 200 iterations, compared to the other algorithms. This might initially seem like a disadvantage, but there is a significant improvement after 200 iterations: despite its slower convergence, the spectral efficiency achieved by MLGWO is significantly higher. Among all the algorithms evaluated, the proposed MLGWO stands out in terms of performance. When assessing spectral efficiency, which measures how efficiently the spectral bandwidth is utilized, the MLGWO outshines the GWO, GJA, PSO, and SMA algorithms. This superior performance indicates that the MLGWO is adept at maximizing the use of available bandwidth, leading to more efficient data transmission. Furthermore, the MLGWO offers another crucial advantage: stability. While some algorithms might exhibit fluctuations or drops in performance, the MLGWO consistently provides stable results. Moreover, it ensures that the minimum rate requirement for the system is met without any significant dips in performance. This stability, combined with its high spectral efficiency, underscores the potential of the MLGWO as a robust and efficient optimization algorithm for the NOMA uplink system.

One of the most insightful plots in this study is the comparison of spectral efficiency against the number of users. This plot is crucial as it demonstrates how the MLGWO algorithm performs as the network becomes denser, which is a common scenario in real-world applications. As the number of users increased, the MLGWO algorithm maintained a high spectral efficiency, indicating its robustness in denser networks. In contrast, other algorithms showed a decline in performance with an increase in the number of users. This is indicative of their inability to handle increased network complexity as efficiently as the proposed MLGWO algorithm. The performance gap between the MLGWO algorithm and others became more pronounced with a higher number of users, emphasizing the scalability of the proposed approach. The comprehensive analysis, including the spectral efficiency against the number of users, has provided compelling evidence of the superiority of the proposed algorithm. Whether it is handling denser networks, achieving faster convergence, or optimizing spectral efficiency, the proposed algorithm consistently outperforms its counterparts. This makes it a promising candidate for real-world applications where network density and efficiency are of paramount importance. In order to compare the computational time of the proposed algorithm and other algorithms, Table 3 is provided with the computational time.

**Table 3:** Computational time of all algorithms for different strategies.

Strategy	Computation time in minutes				
	MLGWO	GWO	PSO	SMA	GJA
Optimal NOMA	3.475	3.211	3.178	3.971	3.593
Sub-optimal NOMA	3.417	3.114	3.076	3.848	3.509
OMA	3.227	3.077	2.974	3.796	3.414

Table 3 compares the computational time of the proposed MLGWO with other state-of-the-art algorithms across different strategies, such as optimal NOMA, sub-optimal NOMA, and OMA. Optimal NOMA requires the highest computational time because it involves the most comprehensive and detailed optimization process. This strategy necessitates precise power allocation and user grouping to maximize spectral efficiency, which involves evaluating a larger solution space and more intricate calculations to achieve near-perfect SIC. Sub-optimal NOMA, on the other hand, takes slightly less time as it simplifies some of the optimization criteria, reducing the complexity of the calculations compared to the Optimal NOMA. While it still aims to enhance spectral efficiency, it employs approximations or less stringent conditions that require fewer iterations and computational resources than the optimal strategy. OMA takes the least computational time since it involves a simpler resource allocation method that does not require the same level of detailed optimization as NOMA strategies. The simpler nature of OMA means fewer calculations and iterations are needed, leading to reduced computational time. The results indicate that while MLGWO exhibits slightly higher computational times per iteration compared to GWO and PSO, it consistently outperforms SMA and GJA. For instance, MLGWO requires 3.475 min for the Optimal NOMA strategy, which is marginally higher than GWO (3.211 min) and PSO (3.178 min) but significantly lower than SMA (3.971 min) and GJA (3.593 min). Despite the marginally higher computational time per iteration, the advanced learning mechanisms in MLGWO, such as competitive learning, Q-learning, and greedy selection, ensure faster overall convergence and superior optimization performance, justifying the slight increase in computational time. These computational times are reasonable given the significant improvements in solution quality and convergence speed achieved by MLGWO, making it a highly effective and efficient algorithm for optimizing spectral efficiency in NOMA uplink systems.

### Further discussions

The MLGWO integrates three mechanisms, as discussed earlier, which effectively balances exploration and exploitation. Competitive Learning maintains diversity by allowing agents to compete and adapt their strategies, preventing premature convergence. Q-learning leverages past experiences to guide the search process, ensuring that the optimizer learns from previous iterations and avoids redundant exploration of suboptimal regions. Greedy Selection retains the best solutions at each iteration, ensuring that high-quality solutions are not lost. This enhanced balance is a significant factor in the superior performance of MLGWO, as it ensures a comprehensive search of the solution space while refining the best solutions. The adaptive learning mechanisms in MLGWO, particularly Q-learning, allow the algorithm to adjust its search strategy based on historical performance data dynamically. This adaptability ensures that the optimizer can respond effectively to the evolving search landscape, improving convergence speed and solution quality. In contrast, traditional methods such as standard GWO, GJA, PSO, and SMA lack such adaptive learning capabilities, making them more prone to getting stuck in local optima or requiring extensive parameter tuning.

The integration of Greedy Selection and Q-learning in MLGWO leads to more efficient use of computational resources. By focusing computational efforts on high-potential solutions and avoiding redundant calculations, MLGWO reduces the overall computational burden. This efficiency is reflected in faster convergence times and lower computational demands compared to other state-of-the-art algorithms, which often involve more complex iterative processes and higher computational overheads. Competitive Learning introduces a mechanism where agents compete based on their performance, ensuring that a diverse set of solutions is explored. This diversity is crucial for avoiding premature convergence and ensuring that the optimizer can explore a wide range of potential solutions. Traditional methods like standard GWO and PSO often suffer from reduced diversity as the search progresses, leading to suboptimal performance. GJA and SMA, while innovative, still struggle with maintaining an optimal balance between exploration and exploitation without the structured competition provided by Competitive Learning. The empirical results demonstrate that MLGWO consistently achieves higher spectral efficiency and lower system complexity compared to standard GWO, GJA, PSO, and SMA. The observed performance improvements are attributed to the algorithm's ability to dynamically adapt its search strategy, maintain solution diversity, and efficiently exploit high-quality solutions. The quantitative metrics, including convergence speed, solution quality, and computational time, provide strong evidence of superior performance.

## Conclusions

The NOMA approach has attracted significant interest lately due to its notable spectral efficiency, positioning it as a key player in enhancing the capacity of future networks. Effective user grouping and a robust power control strategy are pivotal in optimizing the performance of communication systems. The proposed study delves into a combined optimization challenge, focusing on user grouping and power control while ensuring minimum rate requirements. This is done within the context of uplink NOMA transmissions, aiming to strengthen spectral efficiency. A linear programming methodology is employed to handle the power control challenge. Additionally, the task of user pairing and grouping is addressed using five distinct algorithms: proposed MLGWO, GWO, GJA, PSO, and SMA. Simulation results indicate that the achieved spectral efficiency closely matches the optimal solution and surpasses the performance of OMA systems. Notably, the MLGWO algorithm, when applied to this integrated challenge for uplink transmissions, outperforms the traditional OMA in terms of spectral efficiency. Moreover, the suggested MLGWO demonstrates superior results compared to GWO, GJA, PSO, SMA, and other existing algorithms from previous studies. Better performance is achieved while maintaining a reduced system complexity and adhering to constraints specific to uplink NOMA systems. Furthermore, the results highlight that as the user count increases, the combined optimization problem becomes increasingly complicated, demanding more network resources for resolution.

The following future extensions provide a roadmap for continued research and development in the NOMA uplink systems, ensuring that the technology remains cutting-edge and reports the growing demands of communication networks. While the MLGWO algorithm has shown promising results, there is a potential to explore other nature-inspired algorithms or hybrid models that combine the strengths of multiple algorithms. This could further optimize the uplink NOMA transmissions. Machine learning techniques could be integrated to predict user behaviour and network demands, allowing for dynamic adjustments in user grouping and power control, leading to even more efficient resource utilization. As highlighted, the complexity increases with the number of users. Future research could focus on scalability solutions, ensuring that as networks grow, the performance remains optimal. The integration of NOMA with other emerging technologies, such as Massive MIMO or mmWave, could be explored to see how they complement each other in enhancing network performance. With increased spectral efficiency and user grouping, security challenges might arise. Future extensions could delve into ensuring that as performance is optimized, security is not compromised. Alongside spectral efficiency, energy consumption is a critical concern for future networks. Research could be directed towards making NOMA systems more energy-efficient without sacrificing performance. Instead of focusing solely on the physical layer, future research could explore optimizations that span multiple layers of the communication protocol stack, ensuring holistic improvements.

## Data Availability

The datasets used during the current study are available from the corresponding author upon reasonable request.
